# Marine *Actinobacteria* as a source of compounds for phytopathogen control: An integrative metabolic-profiling / bioactivity and taxonomical approach

**DOI:** 10.1371/journal.pone.0170148

**Published:** 2017-02-22

**Authors:** Luz A. Betancur, Sandra J. Naranjo-Gaybor, Diana M. Vinchira-Villarraga, Nubia C. Moreno-Sarmiento, Luis A. Maldonado, Zulma R. Suarez-Moreno, Alejandro Acosta-González, Gillermo F. Padilla-Gonzalez, Mónica Puyana, Leonardo Castellanos, Freddy A. Ramos

**Affiliations:** 1 Universidad Nacional de Colombia, Sede Bogotá, Departamento de Química, Carrera, Edificio de Química of 427, Bogotá, Colombia; 2 Universidad de Caldas. Departamento de Química. Edificio Orlando Sierra, Bloque B, Sede Palogrande Calle. Manizales, Caldas, Colombia; 3 Universidad de las Fuerzas Armadas, ESPE Carrera de Ingeniería Agropecuaria IASA II Av. General Rumiñahui s/n, Sangolquí- Ecuador; 4 Universidad Autónoma Metropolitana Rectoría—Secretaría General, Prolongación Canal de Miramontes, Col. Ex-hacienda San Juan de Dios, Tlalpan, México DF; 5 Investigación y Desarrollo, Empresa Colombiana de Productos Veterinarios VECOL S.A., Bogotá D.C; 6 Universidad de la Sabana, Facultad de Ingeniería, Autopista Norte, Chía, Cundinamarca, Colombia; 7 Universidade de São Paulo, Faculdade de Ciências Farmacêuticas de Ribeirão Preto, Av. do de Sao Paulo, Faculdade de Ciências Farmacêuticas de Ribeirão Preto, Av. do Café, Ribeirão Preto–SP, Brazil; 8 Departamento de Ciencias Biológicas y Ambientales, Programa de Biología Marina, Universidad Jorge Tadeo Lozano, Carrera, Modulo, Oficina, Bogotá, Colombia; Universite Paris-Sud, FRANCE

## Abstract

Marine bacteria are considered as promising sources for the discovery of novel biologically active compounds. In this study, samples of sediment, invertebrate and algae were collected from the Providencia and Santa Catalina coral reef (Colombian Caribbean Sea) with the aim of isolating *Actinobateria*-like strain able to produce antimicrobial and quorum quenching compounds against pathogens. Several approaches were used to select actinobacterial isolates, obtaining 203 strains from all samples. According to their 16S rRNA gene sequencing, a total of 24 strains was classified within *Actinobacteria* represented by three genera: *Streptomyces*, *Micromonospora*, and *Gordonia*. In order to assess their metabolic profiles, the actinobacterial strains were grown in liquid cultures, and LC-MS-based analyses from ethyl acetate fractions were performed. Based on taxonomical classification, screening information of activity against phytopathogenic strains and quorum quenching activity, as well as metabolic profiling, six out of the 24 isolates were selected for follow-up with chemical isolation and structure identification analyses of putative metabolites involved in antimicrobial activities.

## Introduction

During the second half of the twentieth century, one of the major concerns in agriculture was focused on pollution originated by the extensive use of highly toxic agrochemicals such as pesticides [1, 2]. Studies since the 1970s have shown that, besides the harmful effects at the public-health level, the use of pesticides have led to the emergence of phytopathogen resistance caused by the systematic use of a product [3]. As the presence of pathogens in crops of global economic importance is persistent, both industry and academy have increased their efforts in finding solutions to this problem.

Bacterial colonies of both, pathogenic and benefic strains are recognized as social communities that are able to regulate their gene expression in a density-dependent way, phenomenon known as Quorum sensing (QS) [4]. QS systems regulate many processes related to metabolism, development and virulence in bacterial cells, through the production of small signaling molecules, that increase in concentration with increasing cell numbers [5]. Once the concentration of these molecules reaches a specific threshold, different signal transduction cascades are induced resulting in changes in gene expression, including pathogenic effects. The expression of some virulence-related traits are dependent on QS and many plat-pathogenic bacteria are reliant on this kind of systems to evoke disease in its plant host [6, 7]. As an example, it has been widely recognized that toxoflavin production in some *Burkholderia* species (particularly in *B*. *glumae*) is controlled by a QS system and that, the production of this phytotoxin is a key pathogenicity factor in rice rot and wilt [8].

An alternative to solve this problem is the search of antibiotic compounds derived from microorganisms, largely recognized as the main source of antimicrobial compounds, with a potential use against phytopathogens [9]. Microorganisms from terrestrial environments have been the traditional source of these compounds. Marine microorganisms have been recognized as an important source of secondary metabolites with the potential to control these pathogens during the last years [10, 11]. Furthermore, marine representatives of the Phyla *Actinobacteria* are recognized as one of the most important groups with biotechnological potential [12], thus contributing to increasing the supply of new bioactive compounds [13]. Marine-derived metabolites become prototypes for the development of new substances with a putative insecticidal and antimicrobial potential, which make them excellent candidates for their use as agrochemicals [14, 15]. One clear example is the case of Kasugamycin^TM^, a systemic fungicide against *Magnaporthe grisea* and bactericide against *Burkholderia glumae* [16]. This bioactive compound was isolated for the first time from *Streptomyces kasugaensis*, a terrestrial *Actinobacteria*, and later from marine strains of *Streptomyces rutgersensis subsp*. *gulangyunensis* [17, 18].

The mode of therapeutic efficacy of antibiotics is attributed to the inhibition of bacterial growth *in vivo* when antibiotic concentrations exceed the MIC. However, concentrations below the MIC can still attenuate growth and the expression of a variety of bacterial virulence factors, compromising the ability of the pathogen to cause disease. This activity of antibiotics is referred to as sub-MIC effects, and the compounds that produce these effects are known as quorum quenching compounds.

Quorum quenching compounds also have been used in order to inhibit the expression of phytopathogen virulence factors. One of three different strategies can achieve this: the production of enzymes, that degradates the signal molecules; the inhibition of the enzymes (its transcription or its activity) involved in the biosynthesis of the signal-molecule; or the inhibition of the activation receptors of QS [4]. There are some examples of bacterial strains able to inhibit QS systems for pathogenic strains. For example, the expression of lactonase enzyme from *Bacillus* 240B1, codified by the *aiiA* gene, in *Pectobacterium carotovorum* (formely *Erwinia carotovora*) reduce significantly AHL release and soft rot disease symptoms in different detached tissues of potato, eggplant, Chinese cabbage, carrot and celery [19]. In addition, some strains of *Streptomyces* have demonstrated ability to inhibit the expression of different virulence factors regulated by QS in *P*. *caratovorum*, through the production of some secondary metabolites identified as piericidin A and glucopiericidin A, showing that they could have potential use as control agents of some plants pathogens [20]. Compounds derived from marine microorganisms have also been evaluated for their role as quorum quenchers, suitable for acting as anti-pathogenic compounds through interruption of pathogenic bacterial communication; this interruption reduces damage in the host [21, 22]. For instance, the linear dipeptides Pro–Gly and N-amido-α-proline from *Actinobacteria* associated to a marine sponge showed inhibitory activities against quorum sensing (QS) mediated virulence factors in *Pseudomonas aeruginosa* [23].

However, despite the efforts to isolate new compounds from marine environments, traditional bioprospecting approaches including bioassay-guided fractionation often lead to isolation of already known compounds [24]. This, in part, is due to the use of classical approaches for selecting microbial strains based solely on taxonomical or antimicrobial information [25]. Furthermore, studies based exclusively on chemical diversity of compounds lack data on their biological activity, which limits their impact for further applications. For these reasons, bioprospecting of microbial strains for isolating new bioactive compounds has moved towards integrated strategies, which combines phylogenetic data and bioactivity tests with dereplication approaches, as a quick alternative for identifying known and bioactive metabolites in a sample mixture [26]. These strategies, complemented with accurate multivariate analyses (PCA, HCA, OPLS and others), have shown to be effective in identifying new bioactive compounds, improving the pipeline for drug discovery programs using microorganisms [27].

In this study, we present a strategy to study the metabolic potential of selected strains of marine *Actinobacteria* from the Colombian Caribbean Sea, based on the integration of taxonomical information; screening data of antimicrobial activities against phytopathogenic strains (*Burkholderia plantarii* ATCC 43733, *Burkholderia glumae* ATCC 33617, *Burkholderia gladioli* 3704-1-FEDEARROZ-). For antifungal tests, phytopathogenic fungi *Fusarium oxysporum* f. sp. *dianthi* race 2, *Colletotrichum gloeosporioides* 26B and quorum quenching activity (*Chromobacterium violaceum* ATCC 31532) were used, along with their HPLC-MS metabolic profile. All the data sets obtained here were integrated to prioritize selection of isolates for follow-up with chemical isolation and structure identification analyses of active compounds. A discussion on the advantages of the integrative approach and the possible outputs of this strategy is also presented.

## Materials and methods

### Isolation of bacterial strains

In order to conduct this research the ANLA (Autoridad Nacional de Licencias Ambientales) and the Ministerio de Ambiente y Desarrollo Sostenible granted permission to collect samples and study the recovered bacteria (Permission N° 4 of 10/02/2010, Anexo 2, Contrato de Acceso a Recurso Genético No 108). For bacterial strains isolation, small pieces (2 cm^2^) of marine invertebrates, algae and sediments were collected by SCUBA diving at reefs at Old Providence Island (Colombian Caribbean Sea). The geographic coordinates of sampling area was 13°23'0,00"N a 13°25'0.00"N 81°22'0.00"O to 81°24'0,00". Samples of the same substrates were collected in duplicate at five different sites on March of 2013. Fragments of gorgonians and algae were cut off with sharp scissors. Sponge fragments were cut off with a diving knife. Sediment samples (5 cm3) were collected using plastic spoons. Each sample was individually placed in a small ziplock bag and brought to the surface. Samples were gently rinsed with sterile water and then placed directly on the surface of solid culture media plates.

The recovery of actinobacterial strains was performed by using enriched media such as ISP-2 agar (yeast extract 4g/L, malt extract 10 g/L, dextrose 4 g/L, agar 20 g/L) and oatmeal agar supplemented with salts (oatmeal 30 g/L, NaCl 20.8 g/L, KCl 0.56 g/L, MgSO_4_ 4.8 g/L, Rila sea salt 1.5 g/L, glycerol 1.5 g/L, agar 18 g/L). Plates were incubated for two weeks at 30°C.

Once bacterial growth was detected, agar was fragmented into pieces with a sterile needle. The obtained fragments were transferred to 2.0 mL of sterile saline solution (0.85%) and then homogenized in a vortex and supplemented with 50 mg of CaCO_3_ in order to improve the growth of *Actinobacteria* strains. Such suspensions were incubated at 26°C for one week and then serially diluted with Tween-80® (0.1%). Dilutions from 10^−1^ to 10^−5^ were massive cultivated in casein-starch agar (CSA) (Casein 1 g/L, Starch 10 g/L, K_2_HPO_4_ 0.5 g/L Agar 15 g/L) and incubated at 26°C for a period of 4 to 6 weeks.

Purification of bacterial isolates was performed by successive sub-culture of single colonies in CSA medium. A total of 203 isolates was recovered, including, 162 bacteria and 41 fungi. Bacterial isolates were preserved as follows: i) For the bacteria forming mycelium (typical of *Actinobacteria*-like organisms), single colonies were grown in CSA plates at 4°C (short term preservation) and mycelia suspensions were preserved with glycerol (50%) at -80°C (Long term preservation) [28]. ii) For those bacteria with no mycelia formation, a single colony was grown in tryptic soy agar (Difco), and a loopful of biomass was suspended in Tryptic Soy Broth supplemented with glycerol 50%, which were then stored at -80°C. Taxonomical identification, biochemical characterization and metabolic profiling studies were done to 24 strains due to their microscopic and macroscopic characteristics as *Actinobacteria*-like strains (*vide infra*). All the strains were registered in the collection of microorganisms of the IBUN (Instituto de Biotecnología—Universidad Nacional de Colombia—Sede Bogotá).

### Biochemical characterization of bacterial strains

Single colonies of the *Actinobacteria*-like strains were characterized by their colony morphology in nutrient agar, ISP-2, ISP-3, and ISP-4 (S1 Fig). Catalase and oxidase (Bactident ® Oxidasa MERKC) activity were tested as suggested for *Streptomyces*-like bacteria [29, 30]. Other biochemical characteristics such as fermentation or oxidation of sugars (glucose, manitol, inositol, sorbitol, rhamnose, sucrose, melibiose, amygdalin and arabinose); enzyme activity (β-galactosidase, arginine-dihydrolase, lysine decarboxylase, ornithine decarboxylase, ureasae, tryptophan deaminase, gelatinase); citrate utilization; production of sulfide, indole and acetoin; and nitrate reduction were tested with the API 20E kit (bioMérieux Inc., Durham, NC) (S1 Table). The results were compared with those described for other *Actinobacteria*-like bacteria [31].

For a chemotaxonomic characterization of the *Actinobacteria* strains, the presence of L,L-diaminopimelic acid (LL-A_2_pm) or meso- diaminopimelic acid in the cell wall of the isolates was determined through the protocol established by Staneck and Roberts [32] with some modifications [33]. Briefly, two or three loopfuls of fresh biomass (7–14 days growth) were suspended in 500 μL of HCl 6N. Such suspension was homogenized in a vortex for 10 min and then placed in an oven at 100°C for 8 h. After centrifugation, spent supernatants were transferred to a new Eppendorf tube, and dried at 100°C. 500 μL of sterile distilled water were added to each tube, and samples were centrifuged and dried once more, until supernatants were completely dried. This material was the suspended into 75 μL of distilled water, and 3 μL of each sample used for further TLC analyses and 1 μL diaminopimelic acids mixture (CHEM-IMPEX INT´L INC, 1% in distilled water) were loaded on cellulose TLC plates 20x20. TLC plates were developed in saturated glass chambers containing methanol-water-6N HCl-pyridine (80:26:4:10, v/v). The plates were dried, sprayed with a freshly prepared ninhydrin solution (0.2%, w/v dissolved in acetone) and heated at 100°C for 5 min. Presence of L,L-diaminopimelic acid was detected in those regions showing a characteristic olive green color (Rf = 0.6). (S2 Fig).

### Scanning Electron Microscope (SEM) analysis

Scanning electron micrograph analysis strains were growing for 8 days on ISP-2 agar medium using filter paper. Cultures of strains are fixed using Milloning phosphate buffer (0.1 M, pH 7.2) Glutaraldehyde (1%). The cells were rinsed with distilled water subsequently dehydrated with ethanol. The filter paper was first dried, then fixed onto stubs and coated with graphite. The scanning microscopy analysis was performed using SEM-Tescan Vega 3 SB [34, 35].

### Taxonomical identification and accession numbers

24 isolates were selected for further studies based on their microscopic and macroscopic characteristics consistent with *Actinobacteria*-like strains. For the taxonomical identification of the selected strains, PCR amplification and sequencing of the entire 16S rRNA locus were done by using universal primers 27F and 1492R, as previously described [36]. Sequences were trimmed and assembled with DNA Baser software 2.9 and an accurate alignment was obtained using the SINA online tool [37]. Neighbor-joining trees of isolates and their closest relatives were generated using the ARB software [38] and the LTPs123 available in the SILVA database [39]. All DNA 16S rRNA nucleotide sequences for the 24 strains were deposited in GenBank/EMBL/DDBJ, under the accession numbers KX641378 to KX641401, as presented in Table 1.

**Table 1:** Affiliation of *Actinobacteria* strains isolated from marine samples.

Cluster (OTU)	Closest neighbors	Sim %	Strain number	Strain/ GenBank accession numbers	Source
**1**	***Streptomyces griseochromogenes***	**99.86**	**3**	**KX641378**	*Niphates digitalis* (Porifera)
			**6**	**KX641383**	*Niphates digitalis* (Porifera)
	***Streptomyces violascens***	**99.86**	**13**	**KX641381**	Sediment
			**87**	**KX641385**	*Niphates digitalis* (Porifera)
	***Streptomyces resistomycificus***	**99.86**	**89.3**	**KX641386**	*Niphates digitalis* (Porifera)
			**89.4**	**KX641387**	*Niphates digitalis* (Porifera)
	***Streptomyces hydrogenans***	**99.86**	**161a**	**KX641396**	*Bryopsis sp*. (Chlorophyta)
			**161b**	**KX641397**	*Bryopsis sp*. (Chlorophyta)
	***Streptomyces albidoflavus***	**99.86**	**184**	**KX641399**	*Eunicea fusca* (Gorgonacea)
			**194**	**KX641400**	Sediment
			**208**	**KX641401**	*Eunicea fusca* (Gorgonacea)
**2**	***Streptomyces sp*. *MBRL172***	**99.03**	**182**	**KX641398**	*Amphiroa sp*. (Rhodophyta)
**3**	***Streptomyces sanyensis***	**99.72**	**5**	**KX641379**	*Niphates digitalis* (Porifera)
			**143**	**KX641389**	*Niphates digitalis* (Porifera)
			**144**	**KX641390**	*Niphates digitalis* (Porifera)
			**144a**	**KX641391**	*Niphates digitalis* (Porifera)
			**145**	**KX641392**	*Niphates digitalis* (Porifera)
			**148**	**KX641393**	*Niphates digitalis* (Porifera)
			**149**	**KX641394**	*Niphates digitalis* (Porifera)
			**149a**	**KX641395**	*Niphates digitalis* (Porifera)
**4**	***Streptomyces microflavus***	**99.86**	**46b**	**KX641384**	*Codium sp*. (Chlorophyta)
	***Streptomyces fulvorobeus***	**99.86**			
**5**	***Streptomyces pratensis***	**100**	**9**	**KX641380**	*Dictyota sp*. (Phaeophtyta)
**6**	***Micromonospora chalcea***	**99.79**	**102N**	**KX641388**	*Dictyota sp*. (Phaeophtyta)
	***Micromonospora maritima***	**99.38**			
	***Micromonospora sediminicola***	**99.15**			
	***Micromonospora marina***	**99.44**			
**7**	***Gordonia bronchialis***	**98.41**	**25**	**KX641382**	*Xestospongia sp*.(Porifera)

Selected morphotypes were identified in parallel to 16S rRNA approach by mass spectrometry with MALDI-TOF (Matrix-assisted laser desorption/ionization time of flight) Biotyper BRUKER. For this purpose, strains were re-growth in LB media and incubated for eight days at the same temperatures. Identification was done by direct smear method, where a single colony was picked and placed onto a MALDI target plate as a thin layer and allowed to dry at room temperature. The MALDI plate was subsequently overlaid with 1 μL of 70% formic acid and then covered with the matrix solution (α-cyano-4-hydroxycinnamic acid—HCCA) and air dried. The profiles were visualized with the software FlexControl (version 3.0) and the MALDI Biotyper RTC. Dendograms were obtained using BioTyper software 3.0. For calibration and as a positive control, we used a Bacterial Test Standard (BTS) *Escherichia coli* (DH5α) (Bruker Daltonik GmbH, Bremen, Germany) proteic profile.

### Bioassays using marine bacteria strains

#### Antimicrobial assays

Antibacterial activity from 24 Actinobacteria-like isolates was first tested against 3 bacterial rice pathogens by using a direct confrontation assay. The bacterial pathogens strains included: *Burkholderia plantarii* ATCC 43733, *Burkholderia glumae* ATCC 33617, and *Burkholderia gladioli* CIAT 3704–1, kindly provided by FEDEARROZ, which were isolated from symptomatic rice plants of bacterial panicle blight and rice seedling blight. These bacterial strains were recovered and grown as previously described [40–43]. The confrontation assay was performed as follows: single colonies of each *Actinobacteria*-like isolates were massively streaked on Mueller-Hinton agar plates covering half of the surface, and plates were incubated at 28°C for 10 days. Pathogenic *Burkholderia* where then streaked in a horizontal line, perpendicular to the actinobacterial growth area. Plates were incubated at 37°C and growth of *Burkholderia* strains was daily monitored for three days. *Actinobacteria*-like isolates able to induce total or partial growth inhibition of the pathogens after 48 hours were considered as positive for antibacterial activity.

Antifungal activities from all 24 strains were measured as described by Kanini et al. [44]. For antifungal tests, phytopathogenic fungi *Fusarium oxysporum* f. sp. *dianthi* race 2, isolated from vascular wilt symptomatic carnation plants [45], and *Colletotrichum gloeosporioides* 26B, obtained from yam plants with classical symptoms of anthracnose [46] were tested. These phytopathogenic fungi were kindly provided by the “Grupo de Estudio en Ñame” from the Biotechnology Institute, and the “Estudio de Actividades Metabolicas Vegetales” research group of the Chemistry Department from the Universidad Nacional de Colombia.

#### Quorum quenching assay of marine strains

In order to test the ability of the 24 *Actinobacteria*-like isolates to disrupt the quorum sensing systems, *Chromobacterium violaceum* ATCC31532 was used as a biosensor by means of a cross streak antagonistic assay [43, 47, 48]. Briefly, LB agar plates were seeded with each *Actinobacteria*-like isolate by streaking them in a horizontal line, near to one of the extremes of the agar plate. Then, the biosensor was seeded perpendicularly to this line and incubated for 48 hours. The growth of the *C*. *violaceum* strain without violacein production (white colonies) was regarded as a quorum quenching positive result, while the growth of purple colonies of *C*. *violaceum* or the absence of growth of the biosensor were regarded as a negative effect.

#### Marine bacteria strains culture and extraction

Isolates were recovered from their glycerol stock into ISP-2 plates incubated at 28°C. Strains were subsequently cultured in 100 mL by triplicate in Trypticase Soy Broth (TSB Bacto ^TM^ BD) medium in 500 mL conical flasks by shaking at 130 rpm at 25°C for 2 weeks. Cultures were then centrifuged and filtrated by 0.22 μm, and their spent supernatants were extracted three times with ethyl acetate (EtOAc). Organic extracts were concentrated under vacuum and kept frozen until their use for bioactivity screening (antibacterial, antifungal and quorum quenching activity) and for LC-MS analysis. An EtOAc extract of the culture media TSB was used as blank for both bioassays and chemical analysis.

#### Bioassay using organic extracts

**Antibacterial activity of the extracts: A**ntibacterial activity of the extracts against *Burkholderia* pathogens strains was evaluated by a diffusion test in 96-well microtiter plates [49]. Single colonies of *Burkholderia* spp. were grown in liquid medium King B (KB) at 30°C prior to use. 30 μL of each bacterial culture were then diluted into 200 μL of KB agar medium, in 96 well microtiter plates. 500 μg of each EtOAc extract were then suspended into 30 μL of DMSO 5%, and added into each well. Plates were statically incubated for 24 h at 37°C, and the growth of each *Burkholderia* culture was monitored after 24 h by visual inspection; those wells showing inhibition of *Burkholderia* growth were considered positive for the presence of antibacterial compounds. Gentamicin 0,2 μg/mL was used as positive control for bacterial inhibition, and DMSO 5% and TSB were used as negative controls.

**Antifungal activity of the extracts:** Antifungal activity was also tested using EtOAc extracts obtained for each *Actinobacteria*-like isolate in a 24-well microtiter plate assay. Briefly, each fungal strain was grown in PDA plates, and a conidial suspension was prepared with 0.85% NaCl solution. Then, this conidial suspension was used to inoculate each well, previously filled with 2 mL of PDA. Simultaneously, 500 μg of each EtOAc extracts were dissolved in 30 μL 5% DMSO, and this solution was added to the surface of each well, previously inoculated with the conidial suspension. The plates were then incubated at 26°C for 96 h. Total or partial inhibition of the fungal growth was regarded as positive for the presence of antifungal compounds in such an EtOAc extract. A solution of 1% clotrimazole (5 μL/well) was used as positive control, and 5% DMSO and TSB were used as negative controls.

**Quorum quenching activity of the extracts:** Organic extracts of marine strains were also tested for the presence of quorum quenching compounds, by using a disc-diffusion plate assay [50]. AHL-producing strain *Chromobacterium violaceum* ATCC31532 was used as a biosensor, and 5 mm diameter discs were loaded with 300 μg of each EtOAc fraction. Discs were subsequently placed on the surface of agar plates previously inoculated with a bacterial suspension of *C*. *violaceum* ATCC 31532 (OD _600nm_ 0.5). Plates were incubated for 24 h at 30°C. As negative and positive controls, 300 μL of DMSO (5% v/v) and 200 μg 4-hydroxybenzaldehyde (PHB) per disc were used, respectively. Presence of quorum quenching compounds was identified by the lack of violacein production around the discs [51]. In contrast, whenever zones of growth inhibition were observed, this result was reported as antibacterial extract.

### Metabolic profiling analysis of marine strains

#### Sample preparation and HPLC-MS analysis

Organic extracts for each isolate and medium blank were prepared at a concentration of 1 mg/mL in MeOH. Liquid chromatography-mass spectrometry experiments were carried out in an HPLC LaChrom (VWR HITACHI-ELITE) coupled to ESI-IT mass spectrometer Amazon X (Bruker-Daltonics). The chromatographic analysis used a XTerra C-18 column (4.6 × 150 mm, 5μm) and a flow rate of 1.0 mL/min. The mobile phase consisted of 0.075% formic acid (A) and acetonitrile with 0.075% formic acid (B). The solvent program started with 10% B for 3 min and a linear increase to 40% B in 17 min, followed by a linear increase to 100% B in 15 min, then followed by 10 min at 100% B. The total analysis time for each sample was 50 min. The sample (20 μL) was injected by an autosampler. The mass spectrometry was carried out in both positive and negative ionization modes with a spray voltage at 4 kV and capillary temperature at 250°C. The mass range was set from m/z 70–3000. Raw data files generated by HPLC-MS were first converted to netCDF files.

#### Data analysis

The netCDF files were then imported to MZmine 2.10 software [52] for preprocessing steps. Peak detection in MZmine was executed following noise removal, chromatogram construction, and peak deconvolution steps [53]. First, the mass values were detected using the centroid mode in each spectrum and the peaks below 1×10^5^ of the height were discarded as noise. In the second step, chromatograms were constructed for each of the mass values, which span over a certain time range. The minimum time span over the same ion was set as 1 min and the error of the ion *m/z* value was allowed within 100 ppm. The minimum intensity of the highest data point in the chromatogram was set at 1×10^5^. Finally, a deconvolution algorithm was applied to each constructed chromatogram of each mass ion to recognize the actual chromatographic peaks. The “base line cut off” algorithm, which searches for local minima in the chromatogram and separates individual peaks at minimal points was used. The separated peaks were then deisotoped using the function of isotopic peaks grouper in which we set *m/z* tolerance at 1 *m/z*; retention time tolerance at 1 (min); maximum charge of 3; and representative isotope being the most intense. The retention time normalizer was also used after deisotoping to reduce inter-batch variation by setting *m/z* tolerance at 1 *m/z*; retention time tolerance at 1 absolute (min), and minimum standard intensity at 1.1 × 10^5^. Selected MZmine parameters were chosen according to the characteristics of the LC-MS system (e.g. resolution, etc) and experimental results (retention time and m/z deviation). The remaining peaks in different samples were aligned based on the mass and retention time of each peak. The ion *m/z* tolerance for alignment was 1.0 or 100 ppm, retention time was 1 absolute (min), and weight for *m/z* was 20 and Rt was 15 respectively. Following alignment, the resulting peak list was gap-filled with missing peaks using the intensity tolerance of 20% and retention time tolerance of 1 min. The aligned peak list was then exported as a comma separated value (.CSV) file. Data were imported to SIMCA-P 14 (Umetrics, Sweden) for further multivariate data analysis [54]. Barcoding was generated in Microsoft Excel using the IF function (= IF (cell>0,1,0)). This presence-absence created a binary dataset for each bacterial extract showing the overall chemical diversity. Values of “1” are shown as black, while values of “0” are white [27]. Additionally, an in-house developed Excel tool was used to sort and remove features (pair of *m/z* ratios and retention times) associated with cultured media blank (TSB). Dereplication process was done using AntiMarin natural products database, and isotopic abundance was confirmed in HPLC-MS raw data [54].

## Results and discussions

### Marine bacteria isolation and characterization

Environmental samples collected from the coral reef of Providencia (Colombia, South-west Caribbean Sea) were studied to recover associated microorganisms. Environmental samples were processed by using classical enrichment protocols for isolation of culturable *Actinobacteria*, such as the use of ISP-2 and oatmeal agar culture media. [55]. One week later, once the bacterial growth was detected, both agars were fragmented into pieces and transferred to saline solution supplemented with CaCO_3_, in order to improve the growth of *Actinobacteria* strains [56]. Purification of bacterial isolates was performed by successive sub-culture of single colonies in CSA medium, recovering 203 isolates: 162 were described as bacteria while 41 as fungi. In order to identify *Actinobacteria* strains, the bacteria were morphologically characterized in four culture media (ISP-2, ISP-3, ISP-4, nutrient agar), and biochemical tests for sugar fermentation and oxidation as well as for other enzyme activities were also performed. Determination of L,L-diaminopimelic acids (L,L-A_2_pm) as chemotaxonomic marker for *Streptomyces* was also carried out [57]. A complete description of the obtained results for all these biochemical traits is presented in Table and Fig (S1 Table and S1 Fig). Morphological and biochemical data showed that 22 isolates belong to the genus *Streptomyces* as they contained whole-organism hydrolysates rich in L,L-A_2_pm, and they formed extensively branched substrate mycelia that carried abundant aerial hyphae able to differentiate into chains of spores. This set of isolates was of particular interest because *Streptomycetes* are known to be one of the most prolific sources of secondary metabolites [58, 59]. The other two isolates (25 and 102N) showed to contain meso-A_2_pm (S2 Fig), which indicate that they belong to different genera, and it needs the 16S rRNA to be established. Overall, results of biochemical profiles obtained by API 20E test in all 24 strains exhibited differences, which could indicate that isolates are not redundant (S1 Table). For example, although strains 9 and 5 showed the same biochemical tests profile, strain 9 was considered different from 5 since its morphology in four different culture media (ISP-2, ISP-3, ISP-4, nutrient agar) was different from that presented by strains 5. In the same sense, it was found that isolates 89.3 and 89.4 are similar in their biochemical profile, and they only differ in their oxidase phenotype. Regarding the catalase test, all strains were catalase positive, except that of 161b. Additionally the SEM of the strains (S3 Fig) show the spore-surface morphology and the characteristic growth of *Actinobacteria*. These procedures allowed us to recover 24 *Actinobacteria* isolates out of the 162 bacterial isolates (c.a. 15%) obtained from marine sources.

**Fig 1:**
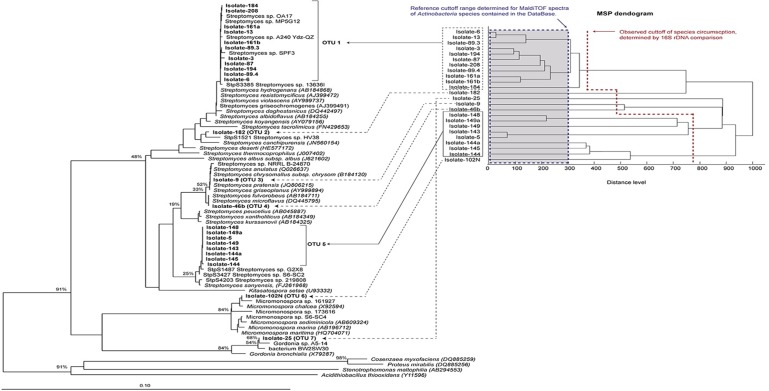
Comparison of a phylogenetic reconstruction based on 16S rRNA genes of the actinobacterial isolates and MALDI-TOF profiles clustering. A Neighbor Joining phylogenetic tree based on 16S rRNA genes of the actinobacterial isolates and their closest relatives (cultivable and uncultivable) was done (left side). Bootstrap calculation was done (1000 repetitions) and is show for the main branches in the tree. A dendrogram based on MALDI-TOF profiles of each isolate (right side) was calculated to compare the obtained results by these two methodologies of classification.

16S rRNA analyses presented in Table 1 and Fig 1 (each> 1400 bp in length) suggested that all 24 isolates were phylogenetically affiliated to phyla *Actinobacteria*, representing three different genera namely *Streptomyces* (22 strains), *Gordonia* (one strain, number 25) and *Micromonospora* (one strain, number 102N). These results are in agreement with the biochemical profiles obtained from API 20-E, which suggest that their phenotype is consistent with their 16S rRNA genotype. Further studies will be carried out in order to determine the precise taxonomical identification to the species level. The majority of the *Actinobacteria* are free-living organisms that are widely distributed in both terrestrial and marine ecosystems, the genera of this phylum exhibit enormous diversity in terms of their morphology, physiology, and metabolic capabilities. The producing capacity of individual *Actinobacteria* can also vary enormously. Some *Streptomyces* species produce a single antibiotic, while others produce a range of different compounds and compound classes [60].

The closest neighbors and similarity percentage for each strain are presented in Table 1. The phylogenetic tree based on 16S rRNA locus for the 24 *Actinobacteria* isolates presented in Fig 1 shows seven operational taxonomical units (OTUs). A total of eleven *Streptomyces* was forming the first clade (OTU 1), composed mainly of related strains recovered from five different sources (Table 1), suggesting the ubiquity of the cluster, which is not considered as a redundant parameter. Its closest neighbors have been recovered mainly from soil. Only *S*. *albidoflavus*, recovered mainly from sea and also soil exhibits both, antifungal and antibacterial activities [61, 62]. Two close neighbors, *S*. *violacens and S*. *resistomycifus* have shown antibacterial activity. Srains of *S*. *violascens* have been reported to produce antibiotics such as actinomycin [63]. The other neighbors *S*. *griseochromogens and S*. *hydrogenans* have shown antifungal activity [64, 65]

Strains 5, 143, 144, 144a, 145, 148, 149 and 149a were grouped within the same cluster (OTU5). The closest neighbor to this group was *Streptomyces sanyensis*. This strain has been recovered from mangrove sediments and has this is its first report of its antimicrobial activity [66, 67]. All these eight strains were recovered from the same sponge *Niphates digitalis*. However, several differences observed between their biochemical profiles and their morphologies in differential ISP media strongly indicates that they should be considered as different isolates. Other five isolates were grouped in five single clusters (i) Strain 182 *Streptomyces* sp. (OTU 2), which is closely related to *Streptomyces canchipurensis* [68]; (ii) Strain 9 (OTU 3), which groups together with *Streptomyces pratensis*, this strain has been recovered from terrestrial environments and there are not reports for its biological activity [69]; (iii); Strain 46b (OTU 4), which is closely related to *Streptomyces microflavus* and *Streptomyces fulvorobeus*. *S*. *microflavus* has been reported from sea and soils, while *S*. *fulvorubeus* has been reported only from the sea [70]. Neither of these two strains have antimicrobial activity reports (iv) Strain 102N (OTU 6) filiated to *Micromonospora* sp. as the closest neighbor; *Micromonospora* species are widely distributed in nature, living in different environments. They have long been known as a significant source of secondary metabolites and it was recently demonstrated that *Micromonospora* species may also influence plant growth and development. The genus has also been reported to produce a large number of antibiotics and is second only after *Streptomyces* in this respect, synthesizing up to 500 different molecules with diverse structures and properties. *Micromonospora* species can also produce hydrolytic enzymes, which allows them to play an active role in the degradation of organic matter in their natural habitats [71]. and (v) strain 25 (OTU 7) with *Gordonia* sp. as the closest neighbor. Members of the genus *Gordonia* were originally isolated as opportunistic pathogens in humans. However, in recent years, most novel members of the genus have been isolated from terrestrial and marine sources [72] Several members of this taxon play important roles in biodegradation and bioremediation. The production of surface-active compounds by *Gordonia* sp. has been associated with their ability to degrade the hydrophobic compounds [73]. Importantly, according to the current limit of circumscription of a prokaryotic species based on the 16S rDNA sequence [74], the isolate 25 could be a putative new species because percentage of 16S rDNA similarity against its closest relative is below 98.7%. In general, we did not find any significant correlation between isolation sources and bacterial species here recovered, which indicates that all sources harbor different *Actinobacteria* species, without apparent recruitment of a particular OTUs by any specific source.

Recently, matrix assisted laser desorption/ionization time of flight mass spectrometry (MALDI-TOF MS) has been used for bacterial dereplication and preliminary identification of environmental isolates. This technique is based upon protein analysis of whole-strain. The observed degree of molecular mass conservation among these proteins renders spectral similarity a suitable marker of phylogenetic relatedness [75]. The MALDI-TOF analysis of 24 *Actinobacteria* strains is shown in Fig 1 and compared to the 16S rDNA analysis. The OTUs clustering observed for 16S rDNA agree with the clustering observed by MALDI-TOF, except for the abundant groups (OTU 1 and 5). The spectra of all actinobacterial species available in the MALDI-TOF database (BDAL) were used to calculate a reference cut-toff that could help us to determine the putative similarity distance that define an actinobacterial species. The calculated cut-off shown that similar species can be defined by 70% of similarity (S4 Fig). When both approaches are compared, MALDI-TOF results suggest that richness of species within OTU 1 and OTU 5 could be higher than the assigned by the 16S rDNA sequence resolution (denoted by the red color). The MSP dendrogram shows a richness of members within the for OTU 1 and confirmed the possible heterogeneity observed among these isolates and described above, probably caused by its distinct isolation origin as commented. On the other hand, the OTU number 5 contains seven *Streptomyces* strains isolated from *Niphates digitalis* as shown by 16S rRNA analysis, and although the isolation source is the same within the isolates of this group, more species richness can be observed compared to OTU 1 where we curiously have more isolation sources.

Previous studies reported that marine Porifera often harbor communities with high diversity levels [53, 63, 76]. Our results show that Porifera *Niphates digitalis* yielded the highest number of *Actinobacteria* strains (13), while *Xetospongia* sp. (1), *Amphimedon compressa* (0), *Plakortis halichondroides* (0) and *Aplysina archeri* (0) showed low diversity in the number of recovered *Actinobacteria* strains. The other environmental sources also showed low diversity: from algae *Dictyota* sp. and *Bryopis* sp. and from octocoral *Eunicea fusca* two *Actinobacteria* strains were recovered, while from the algae *Codium* sp. and *Amphiroa* sp. only one *Actinobacteria* strain could be isolated. In the case of the ten studied sediments, only 2 strains could be recovered. In summary, out of the 23 environmental samples studied, 24 *Actinobacteria* strains could be recovered, including 22 *Streptomyces*, 1 *Gordonia* and 1 *Micromonospora*.

### Bioactivity assays

**Fig 2:**
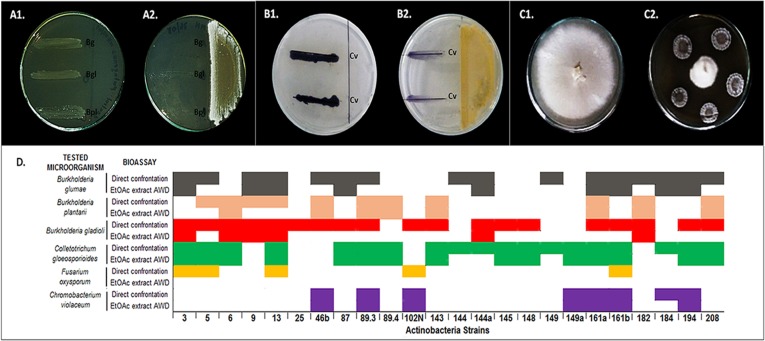
Antimicrobial activity and quorum sensing inhibition tests for bioactivity results of 24 *Actinobacteria* strains. **(A1)** Picture of *Burkholderia* spp. phytopathogens without confrontation (Bg: *Burkholderia glumae*. Bgl: *Burkholderia gladioli*; Bpl: *Burkholderia plantarii*). (**A2)** Picture of confrontation test of *Burkholderia* spp. phytopathogens against strain number 9. (**B1)** Picture of *Chromobacterium violaceum*. (**B2)** Picture of quorum quenching test against strain number 46b. (**C1)**. Picture of *Fusarium oxysporum f*. *sp dianthi* race 2 against strain 5. (**D)** Heat map of bioactivity for the 24 strains and their organic extracts. Results for antibacterial, antifungal and QQ activities are summarized in Fig 2D. Horizontal axis shows the codes of the 24 *Actinobacteria* and vertical axis shows each one of the pathogens tested in both, direct confrontation and extract growth inhibition test. Color indicates the total or partial control of each phytopathogen, assumed as a positive result.

Since no redundancy among isolates could be established, it was decided to include these 24 isolates in further biological activity tests. In order to evaluate the ability of all 24 *Actinobacteria* strains for phytopathogen-growth inhibition (Fig 2), an antagonism test (direct confrontation) was conducted against the bacterial phytopathogens *Burkholderia glumae* ATCC 33617 (causes bacterial panicle blight), *B*. *gladioli* 3704–1 (causes bacterial grain rot and leaf-sheath browning) and, *B*. *plantarii* ATCC 43733 (causes seedling blight in rice). Isolates were also tested for its antifungal activity against phytopathogenic fungi *Fusarium oxysporum* f. sp. *dianthii* (Carnation) and *Colletotrichum gloeosporioides* 26B (Yam).

In addition, as several marine compounds have proved their role as quorum quenchers (QQ) [16], an anti-pathogenic effect that could interrupt bacterial communication reducing damage in the host, the 24 *Actinobacteria* isolates were also tested against *Chromobacterium violaceum* biosensor as an approach to identify strains able to use this mechanism of control of phytopathogens.

Results for some selected *Actinobacteria* strains in antibacterial (Fig 2A), quorum quenching (Fig 2B) and antifungal (Fig 2C) activity tests are presented in Fig 2A–2C. The results for all tests, including the evaluation of the activity for their organic extracts in EtOAc, are summarized in Fig 2D.

The 24 selected strains included in this study showed activity in the direct confrontation test; for some of them maintain their activity in their organic extract. This suggests that phytopathogen growth control was made by secondary metabolite production. For some strains, this activity was observed in the aqueous extract (S5 Fig). However, the high polarity of these extracts makes difficult the isolation of these hydrophilic compounds for further studies. Most extracts keep the same activity observed in direct confrontation tests against. *B*. *plantarii* and *C*. *gloeosporioides*, (8 out of 11 and 15 out of 19 respectively). Similar results were observed in the QQ activity against tests using *C*. *violaceum*, in which 7 out of the 8 strains showed partial or total inhibition of violacein production. Other extracts showed lower or no antibacterial activity in direct confrontation tests against *B*. *glumae* (9/16), *B*. *gladioli* (6/19), and *F*. *oxysporum* (0/5).

In the direct confrontation test against three phytopathogen bacteria, the most active isolates were: strains 5, 9, 13, 46b, 89.3, 161a, 161b, 182 and 208. Against fungi, the most active strains were 3, 5, 13, 161b; and against *C*. *violaceum*, the most active strains were 46b, 89.3, 102N, 149a, 161a, 161b, 184 and 194. In addition, strains 5, 13, 89.3, 161a, and 161.b, showing to be active in five out of the six assays conducted. In this group, all of them were active against *Burkholderia* spp except 161b that does not control *B*. *plantarii*. On the other hand, the least active strain is 25, a *Gordonia* species that only controls *B*. *gladioli*. A poor profile is also observed for the *Micromonospora* strain (Strain 102N), and for *Streptomyces* spp. strains 144 and 149.

In the extract test, the most active one is 161a that shows activity in four out of the five positive direct confrontation assays. This strain extract loses the activity against *B*. *gladioli*. Extracts obtained from strains 3, 13, 89.3, 161b, and 194 are active in three out of the five positive assays previously conducted, meaning that these extracts lose activity against two pathogens. Interestingly, EtOAc extracts from these strains were in most of the cases active against *B*. *glumae* and *C*. *gloeosporioides*. On the other hand, extracts of strains 6, 9, 46b, 87, 89.4, 143, 144a, 149a, 182 and 208 showed to be active in two out of the six assays, mainly against *B*. *plantarii*, and *C*. *gloeosporioides*. Extracts of five strains were active against one pathogen (5, 102N, 145, 148, and 184), and extract of three strains showed no activity (25, 144, and 149).

### Metabolic profiling analysis

**Fig 3:**
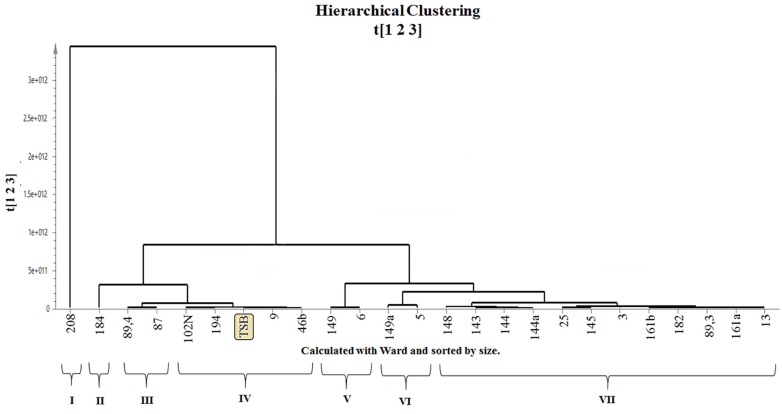
Hierarchical Cluster Analysis (HCA) based on LC-MS metabolic profiles from the organic extracts of 24 bacterial strains and TSB culture media. Arabic numbers represent strain codes. Roman numbers represent clusters according to their metabolic profile similarity.

One of the main goals of this study was to select strains with different chemical profiles for further studies according their metabolic diversity. In order to evaluate such differences in metabolite production, an LCMS analysis of the 24 organic extracts was carried out. Multivariate data analysis (MDVA) allows to simultaneously evaluate a huge number of metabolites and determine their correlations with certain biological properties. Independently from the selected analytical technique, the usually huge number of data used in metabolomics would require multivariate analysis to classify the samples into different groups and to facilitate their interpretation in terms of metabolite distribution under distinct variables. Thus, MVDA applied to LCMS data is required to evaluate chemical richness of the extracts defined as number of compounds present in each extract [54]. Data were exported to an Excel matrix (.CSV) and then preprocessed in MZmine 2.10 free software. For the multivariate analysis, the SIMCA-P 14 statistical package was used. A Hierarchical Cluster Analysis (HCA) was conducted for all 24 EtOAc extracts in addition to the extract of the culture media (TSB), as presented in Fig 3.

The HCA dendrogram of Fig 3 shows that the strains extracts were grouped into seven clusters (I to VII) according to their chemical profile by LCMS. In HCA, the distance measure of two objects corresponds to the degree of similarity between them. Hence, in this case, a small cluster distance indicates a high similarity between the strains chemical profile, and as this distance increases, so does the degree of divergence among them. The TSB blank extract grouped in cluster IV also included strains 9, 46b, 102N and 194, suggesting that the metabolic production of these isolates did not differ substantially from the profile obtained from the culture medium.

The large distance from cluster I (strain 208) relative to the other clusters (cluster II-VII), suggests important differences in terms of metabolic profiles for this strain when compared to all other samples. Considering that strain 208 does not show significant differences to the other strains in terms of bioactivity, biochemical profile and specially in its 16S rRNA sequences, such differences might be explained by the presence of different metabolites probably resulting from the presence of different biosynthetic gene clusters or a differential gene expression. For instance, previous studies have demonstrated that different strains from the same *Streptomyces* species (*S*. *albus*) contain different gene clusters encoding the production of strain-specific secondary metabolites [77]. According to this study, each strain of a *Streptomyces* species likely harbors at least one strain-specific biosynthetic gene cluster [77]. Chemical interaction between strains and their hosts might also help explain differences in the metabolic profile of strain 208, considering that even the same strain interacting with different hosts might show different metabolites.

In the same extent, cluster II comprised a single strain (184), cluster III contains strains 87 and 89.4; Cluster V includes strains 6 and 149; strains 5 and 149a belong to cluster VI. Meanwhile, cluster VII contains a large group of strains 3, 13, 25, 89.3, 143, 144, 144a, 145, 148, 161a 161b, and 182. The fact that HCA does not match neither the phylogenetic reconstruction nor the dendrogram obtained by MALDI-TOF analysis is not surprising as these techniques focus on different sets of information within the bacterial strains, making them complementary. While the phylogenetic tree is based on 16S rRNA sequences similarity, MALDI-TOF is commonly employed for medium to high molecular weight compounds including peptides and proteins. On the other hand, LC-MS focuses on low molecular weight metabolites.

**Fig 4:**
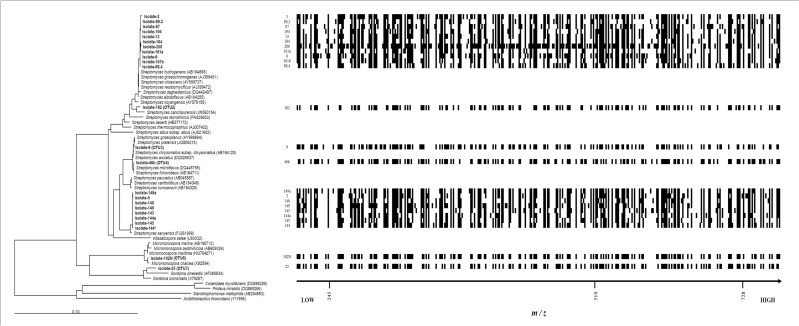
Barcoding vs. Phylogenetic tree. Barcoding based on variables (retention time and *m/z*) displaying distinct metabolic profiles among the 24 *Actinobacteria*. Horizontal axis shows variables expressed as *m/z* values >200 of the 24 extracts. Each variable represents a single compound and its presence is represented by black squares and vertical axis shows each one of *Actinobacteria* strains according to phylogenetic tree.

In order to display the metabolic diversity of the 24 strain extracts, chemical barcoding was built (Fig 4). For this, the CSV file was exported to Microsoft Excel and the data observed for medium blank were removed for each strain datum (only *m/z* values >200 are considered). Then, by using IF function of Excel (= IF (cell>0,1,0)), the presence-absence created a binary dataset (barcoding) for each bacterial extract, showing the overall chemical diversity. Values of presence “1” are shown as solid black color, while values of absence “0” are white. Each variable corresponds to a two-dimension identifier for each compound based on its retention time (LC chromatography) and *m/z* value. By using this barcoding, it is possible to identify some strains with a great metabolic richness (large number of variables), i.e. 208 (236 variables); and some strains with low metabolic diversity such as strains 89.3 and 182 (137 and 129 variables, respectively). This analysis recognized strain 208 as the producer of a large number of exclusive metabolites (35 unique Rt and *m/z* values), thus confirming the HCA results as this strain showed important differences in terms metabolic profile relative to the other samples. Other strains (such as 149a) do not produce exclusive metabolites, because they share all the variables (retention time and *m/z*) with others strains, while strain 102N produced only one unique metabolite (variable) along with other compounds identified in the other extracts here presented.

Orthogonal Partial Least Square-Discriminant Analysis (OPLS-DA) is a supervised MDVA method commonly used to find X variables (e.g., compounds identified by a Rt and *m/z* value in different extracts) correlating with determined Y variables (e.g., antibacterial, antifungal and QQ activity) [78]. After cleaning of the data matrix (which includes removal of the culture medium variables), an OPLS-DA with subsequent identification and dereplication of discriminant variables was performed. In this case, data scaling was performed by unit variance (UV-) scaling, which makes all metabolites become equally important independently of the concentration (opposite to Pareto scaling) [79].

**Fig 5:**
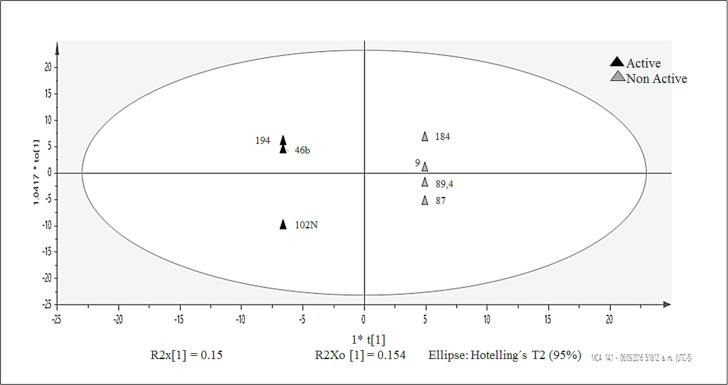
OPLS-DA- Quorum quenching activity. Score Scatter Plot of seven ethyl acetate extracts of strains 9, 46b, 87, 89.4, 102N, 184, 194. Active strain (black color) vs. non-active strain (gray color).

Based on the HCA (Fig 3), two main groups (Group A with clusters II-IV; and Group B with clusters V-VII), besides outlier cluster I (strain 208), were identified. An OPLS-DA using as Y variable, quorum quenching, antibacterial and antifungal activity was performed for each one of the above-mentioned groups. The obtained model for quorum quenching activity applied for Group A (Fig 5) suggested a highly predictive model for this activity (R2Y = 1, Q2 = 0.998), and the VIPs (Variable Influence on Projections) were identified with Loadings-Plot, as presented in S6 Fig.

**Fig 6:**
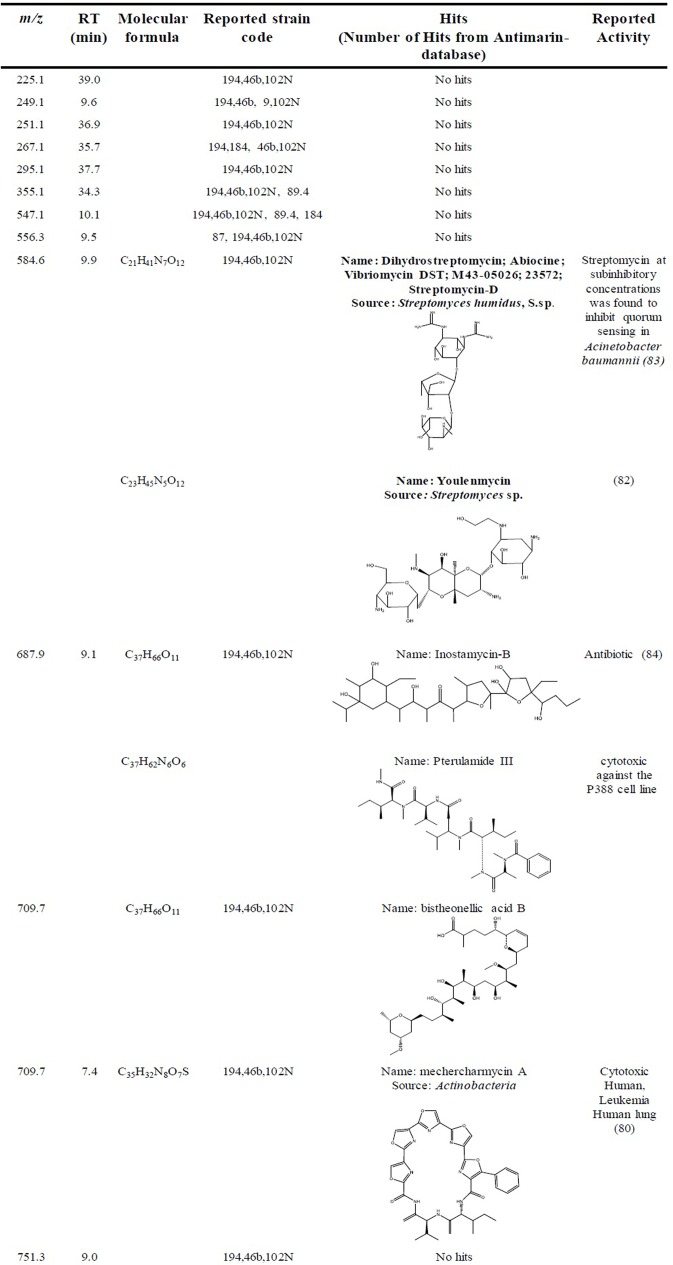
Dereplication. Data for 12 VIPs for quorum quenching activity using Antimarinee database.

It was possible to identify 12 VIPs (compounds) from OPLS-DA-Quorum quenching activity. They represent the quorum quenching active compounds for the 3 active extracts of Group A (cluster II-IV), as follow: Active strain 46b (*Streptomyces* OTU 4), 194 (*Streptomyces* OTU 1) and 102N (*Micromonospora* OTU 6), and inactive strain: 9 (*Streptomyces* OTU 3) and 87, 89.4, 184 (*Streptomyces* OTU 1). Dereplication analysis attempts to reduce time and efforts in isolation and identification of well-known active compounds, or redundant inactive natural products. The dereplication analysis conducted on this OPLS-DA model for quorum quenching activity (Fig 6) showed hits for only 3 out of the 12 found VIPs, as follows: *m/z* 687.9 tentatively identified as Inostamycn-B or Pterulamide III; *m/z* 709.7 tentatively identified as Mechercharmycine B (from unknown *Actinobacteria*) [80]; and *m/z* 584.6, the latter tentatively identified as Streptomycin D (from *Streptomyces* spp) [81] or Youlenmycin (from *Streptomyces* sp.) [82]. Interestingly, Streptomycin D has been reported as QQ in *Acinetobacter baumanii* [83], showing the strengths of this approach to guide isolation efforts on unknown compounds, and the capabilities to identify new sources of known active compounds. The OPLS-DA using quorum quenching activity with group B, antibacterial or antifungal activity with both groups showed to be not confident as predictive models.

### Prioritizing isolates for further chemical studies

Information about taxonomy, biological activity against phytopathogens and metabolic profiling was collected in this study as criteria for strain priorization.

Traditionally, selection of strains for further chemical studies relies only on the bioactivity screening. However, bioactivity data alone do not provide information about chemical compounds responsible for it, and can take to the selection of well-known strains producers of well-known compounds.

Similarly, selection based on phylogeny (16S rRNA sequences) is useful as a guide to select strains from a specific genus, or species, of particular interest, or even to discard redundant isolates or those pathogenic strains from a collection. Nevertheless, this taxonomical data alone does not provide metabolic or bioactivity information. Also, there are examples of phylogenetically related strains which do not produce the same secondary metabolites, and strains belonging to different clades with similar metabolite production [84, 85, 86].

On the other hand, the metabolic profiling use alone showed information about chemical diversity rise as an interesting criterion for strain selection. It is expected that strains able to produce several antimicrobial compounds can contribute to reduce resistance when applied in field treatments for phytopathogen control purposes. However, it should be noted that bacteria producing several compounds (metabolic rich bacteria), would produce low amounts of compounds, making difficult their isolation and identification. Furthermore, differences observed in metabolic profiling could arise from extracts with low diversity of active compounds, suggesting that strains could have developed the ability to produce specifically highly-potent molecules with a small diversity. Additionally, the exclusive use of metabolic profile (LC-MS) alone does not correlate with the desired biological activity.

In general terms, we state that each set of data alone is not enough to make a decision about which strains are suitable to study. To overcome this problem, recently, metabolic profiling analysis based on LC-MS and/or NMR data, together with supervised multivariate analysis such as OPLS-DA that integrates bioactivity information, has been described as a useful tool to obtain information about chemical composition of selected active extracts prior to compound isolation [87–90]. This paper proposes the use of bioactivity data as main criteria, taking into account that a direct confrontation can be used for strain selection in biocontrol studies, but organic extracts provide valuable information to guide further chemical studies. This way, the use of HCA and barcoding refers to metabolic richness, while taxonomical data reflect genetic diversity.

By using this approach, six strains were selected for further chemical studies as a result of integrated bioactivity, metabolic profiling and taxonomical information (Table 2). Strain 161a was selected because it showed the broad spectra of antimicrobial activity with significant differences in its metabolic profile when compared with cluster IV, which contains TSB blank extract, as observed in the HCA analysis. Strain 208 was selected for further chemical analysis because of its very distinctive metabolic profile among all 24 evaluated strains, considered as an outlier in the HCA analysis. It showed to be the most prolific source of unique metabolites in the barcode analysis (236 total variables), despite the fact that they grouped in the same taxonomical OTU 1 together with previous selected strain 161a. However, other strains with broad spectra were not selected. That is the case of strain 194, active against three microorganisms including two pathogens and the QQ biosensor, not sharing the same cluster with 161a. However, HCA data for strain 194 suggest that its metabolic profile is quite similar to that of TSB blank, meaning that recovery of secondary metabolites from its extract could be difficult.

**Table 2:** Strain higher biological activity–HCA- OUT.

Strain	Cluster number (OTU) Phylogenetic analysis	Number of pathogenic organisms controlled	Cluster number (HCA)
161a	1	4	VII
208	1	2	I
5	5	1	VI
9	3	2	IV
182	2	2	VII
102N	6	2	IV

A second group of selected strains comprised those isolates with a selective activity: Strain 5 was selected because of the selective antifungal activity of its organic extract against *C*. *gloeoesporioides*. This strain grouped in cluster VI (HCA) with 149a, and both belong to OTU 5; however, strain 5 showed antifungal activity stronger than 149a (data not shown). Strain 102N, the only member of *Micromonospora* genus (OTU number 6), showed a strong and selective quorum quenching activity. A similar situation can be described for strains 9 (cluster IV) and 182 (cluster VII), which are the unique members of OTUs 3 and 2, respectively.

Finally, dereplication analysis using Antimarin database for strain 208, the most prolific source of compounds, indicated that 28 out of the 36 variables do not present any hits in the database (S2 Table), which could represent novel compounds. The identified metabolites by dereplication process include compounds such as pyridine derivatives (e.g. *m/z* 227.2 tentatively identified as Nikkomycin D from *Streptomyces tendae*, and *m/z* 446.6 tentatively identified as Piericydin-C2 compounds with antibacterial and anthelmintic activity isolated from *Streptomyces piericidicus* and *S*. *pactum* [91, 92]; δ-lactones (e.g. *m/z* 245.3 tentatively identified as Graefe’s Factor I, inducer of anthracycline production isolated from *Streptomyces viridochromogenes*) [93, 94]; fatty acid derivatives (e.g. *m/z* 245.3 tentatively identified as trihomononactic acid, antibacterial from *Streptomyces globisporus*) [95], anthraquinone derivatives (e.g. *m/z* 337.3 tentatively identified as 8-*O*-methyltetrangomycin, with antifungal and antibacterial compound isolated from *Streptomyces* sp.) [96, 97]; alkaloids (e.g. *m/z* 255.2 tentatively identified as *N*-acetylquestiomycin A, antitumor and antifungal from *Streptomyces thioluteus*) [98, 99] and macrolides (m/z 415.1 tentatively identified as Feigrisolide D, antibacterial from *Streptomyces griseus*) [100].

## Conclusions

At this stage, our integrative strategy using taxonomical information, bioactivity and metabolic profiling tools along with dereplication procedures allowed us to select six strains of *Actinobacteria* recovered from marine environments (161a, 208, 5, 9, 182 and 102N), as a source of novel and active compounds against phytopathogens. The preliminary identification of bioactive compounds using dereplication procedures, suggests that these strains are a rich source of compounds, including well known and novel bioactive metabolites. Our data suggest that active strains could act by means of antibiotic (antibacterial and antifungal) or quorum quenching compounds. These strains could be used for biotechnological production of biocontrol agents or for compounds production for agrochemical purposes. This study also contributed to investigate the biodiversity in the southwest Caribbean Sea as a source of unexploited microbial diversity, which has enormous potential to provide chemical compounds to develop novel biotechnological products with possible applications in the field of agriculture and environmental preservation.

## Supporting information

S1 Fig Colony morphology in nutrient agar, ISP-2, ISP-3 and ISP-4. Strain 25 (*Gordonia* sp.), strain 102N (*Micromonospora* sp.) and strain 182 (*Streptomyces* sp.).
(TIF)

S2 Fig L,L-DAP and meso-DAP (diaminopimelic acid) Chemotaxonomic marker in *Streptomyces*. Cellulose plates TLC Merck 20x20 cm. Mobile phase: Methanol: water: 6N HCl: pyridine 80:26:4:10 v%v. Developer: sln 2% ninhydrin.
(TIF)

S3 Fig Scanning electron micrograph of the isolate 5, 182 and 208 growing on ISP-2 agar medium showing spore chain spiral shape and spore surfaces warty. (TIF)

S4 Fig The spectra of all actinobacterial species available in the MALDI-TOF database (BDAL). were used to calculate a reference cut-toff that could help us to determine the putative similarity distance that define an actinobacterial species. The calculated cut-off shown that similar species can be defined by 70% of similarity.
(TIF)

S5 Fig Antimicrobial activity and quorum sensing inhibition tests for Bioactivity results of 24 *Actinobacteria* strains isolated from marine sources. Heat map of bioactivity for the 24 strains and their aqueous extracts. Results for antibacterial, antifungal and QQ activities, are summarized. Horizontal axis shows the codes of the 24 *Actinobacteria* and vertical axis shows each one of the pathogens tested in both, direct confrontation and extract growth inhibition test. Color indicates the total or partial control of each phytopathogen, assumed as a positive result.
(TIF)

S6 Fig OPLS-DA quorum quenching activity. Summary of Fit (R2Y = 1, Q2 = 0.998). Loading Scatter Plot (VIPs, gray color). Strains 9, 46b, 87, 89.4, 102N, 184, 194. In this case data scaling was performed by unit variance (UV-) scaling.
(TIF)

S1 Table Biochemical test for 24 marine strains. **API 20E Test (bioMérieux Inc., Durham, NC)–Oxidase and Catalase–L,L DAP (*Actinobacteria*).** The test shows the presence of: **ONPG**: β-galactosidase; **ADH**: arginine-dihydrolase; **LDC**: lysine decarboxylase; **ODC**: ornithine decarboxylase. **CIT**: citrate utilization. Production of: **H**_**2**_**S**, **URE**: Urease, **TDA**: tryptophan deaminase, **IND**: indole, **VP**: acetoin, **GEL**: gelatinase. Other biochemical characteristics such as fermentation or oxidation of sugars (**GLU**: glucose, **MAN**: manitol, **INO**: inositol, **SOR**: sorbitol, **RHA**: rhamnose, **SAC**: sucrose, **MEL**: melibiose, **AMY**:amygdalin and **ARA**:arabinose It also shows the reduction of nitrates to nitrites.
(PDF)

S2 Table Dereplication data of 36 variables of Strain 208 (Antimarin database). (PDF)
