# Supplementary figures and images for: Marine Actinobacteria as a source of compounds for phytopathogen control: An integrative metabolic-profiling / bioactivity and taxonomical approach
Source: PLoS One. 2017 Feb 22;12(2):e0170148. doi: 10.1371/journal.pone.0170148 (PMC5321270; doi:10.1371/journal.pone.0170148)

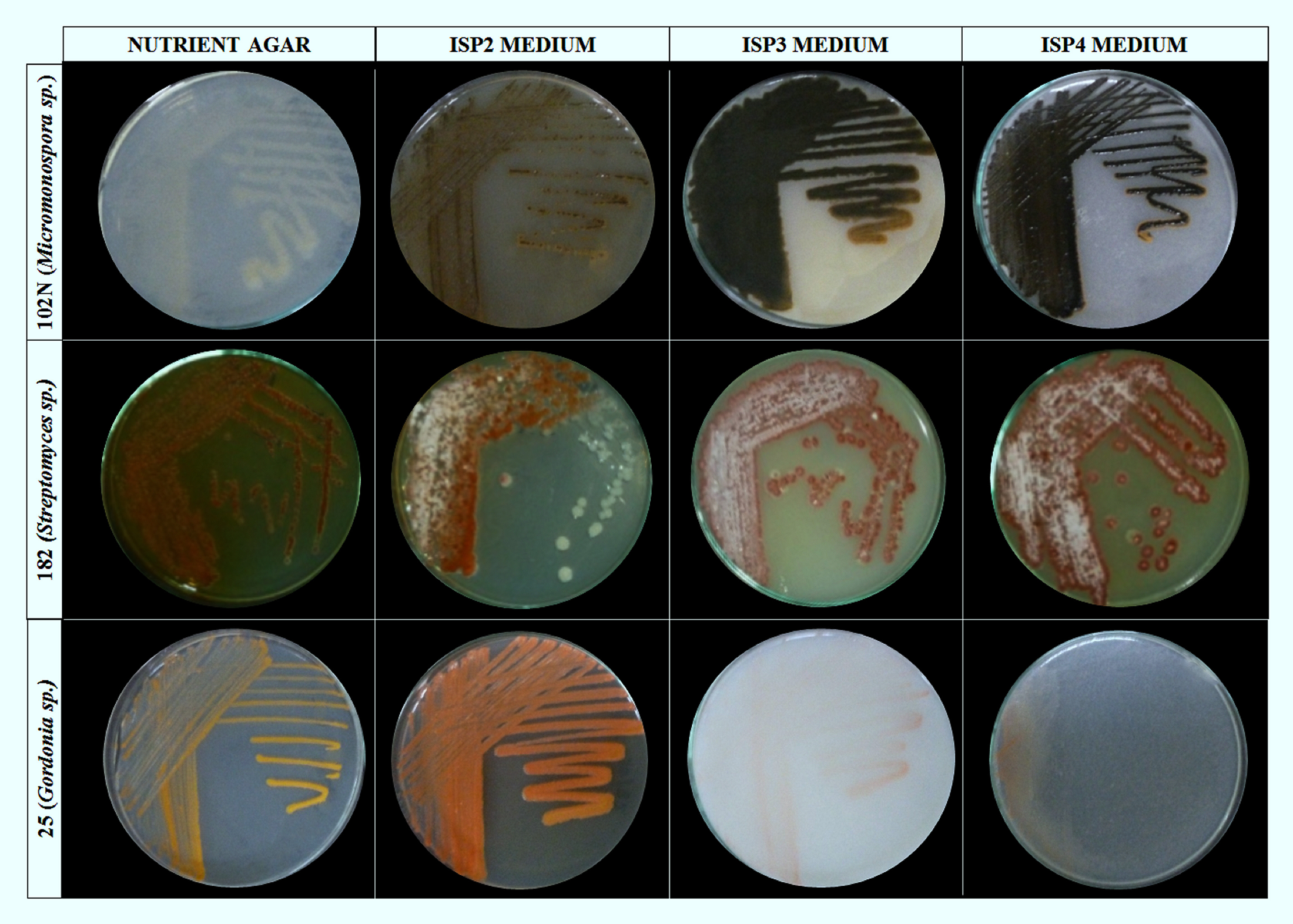

Supplement: S1 Fig — Strain 25 (Gordonia sp.), strain 102N (Micromonospora sp.) and strain 182 (Streptomyces sp.). (TIF) [file pone.0170148.s001.tif]

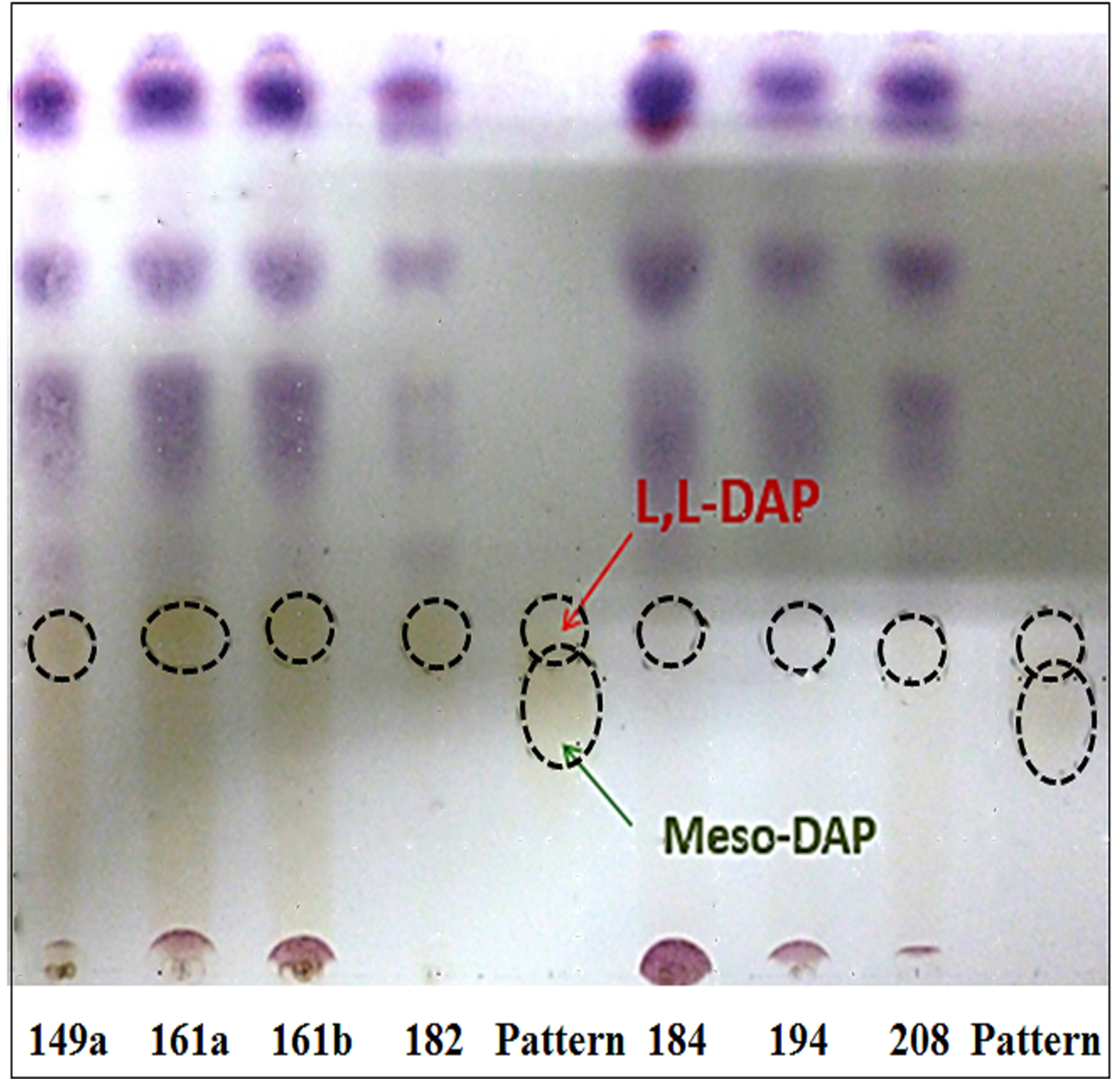

Supplement: S2 Fig — Cellulose plates TLC Merck 20x20 cm. Mobile phase: Methanol: water: 6N HCl: pyridine 80:26:4:10 v%v. Developer: sln 2% ninhydrin. (TIF) [file pone.0170148.s002.tif]

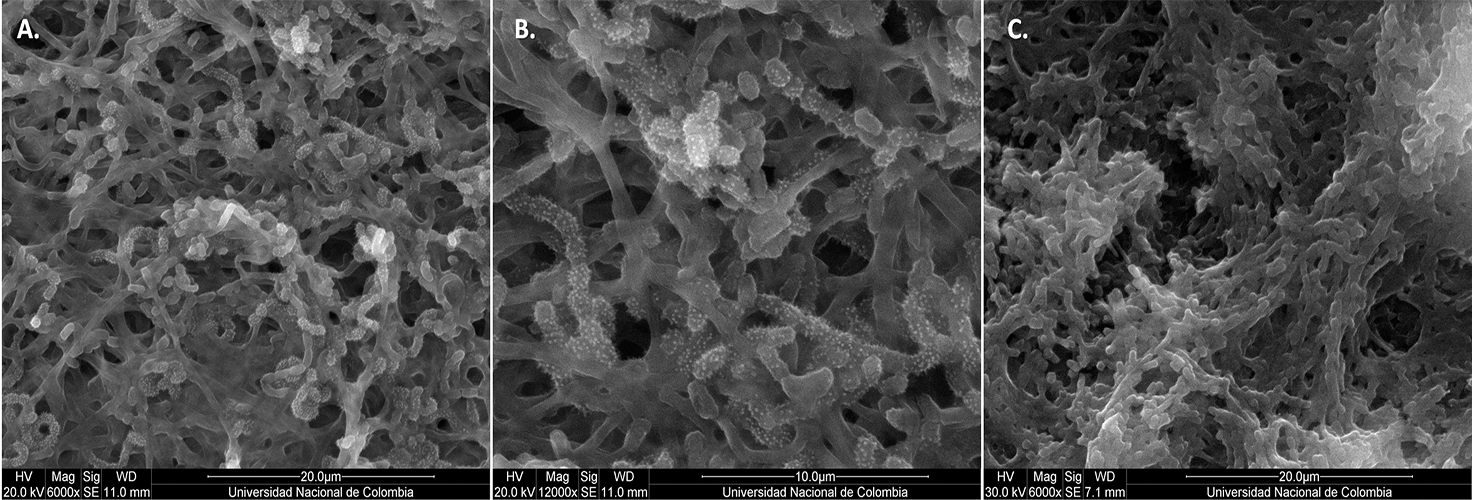

Supplement: S3 Fig — (TIF) [file pone.0170148.s003.tif]

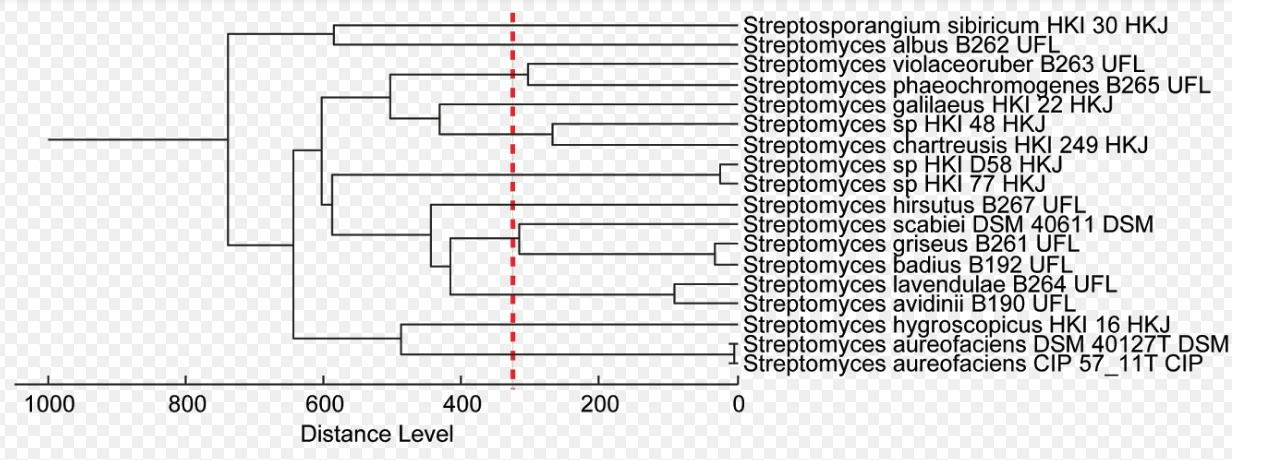

Supplement: S4 Fig — were used to calculate a reference cut-toff that could help us to determine the putative similarity distance that define an actinobacterial species. The calculated cut-off shown that similar species can be defined by 70% of similarity. (TIF) [file pone.0170148.s004.tif]

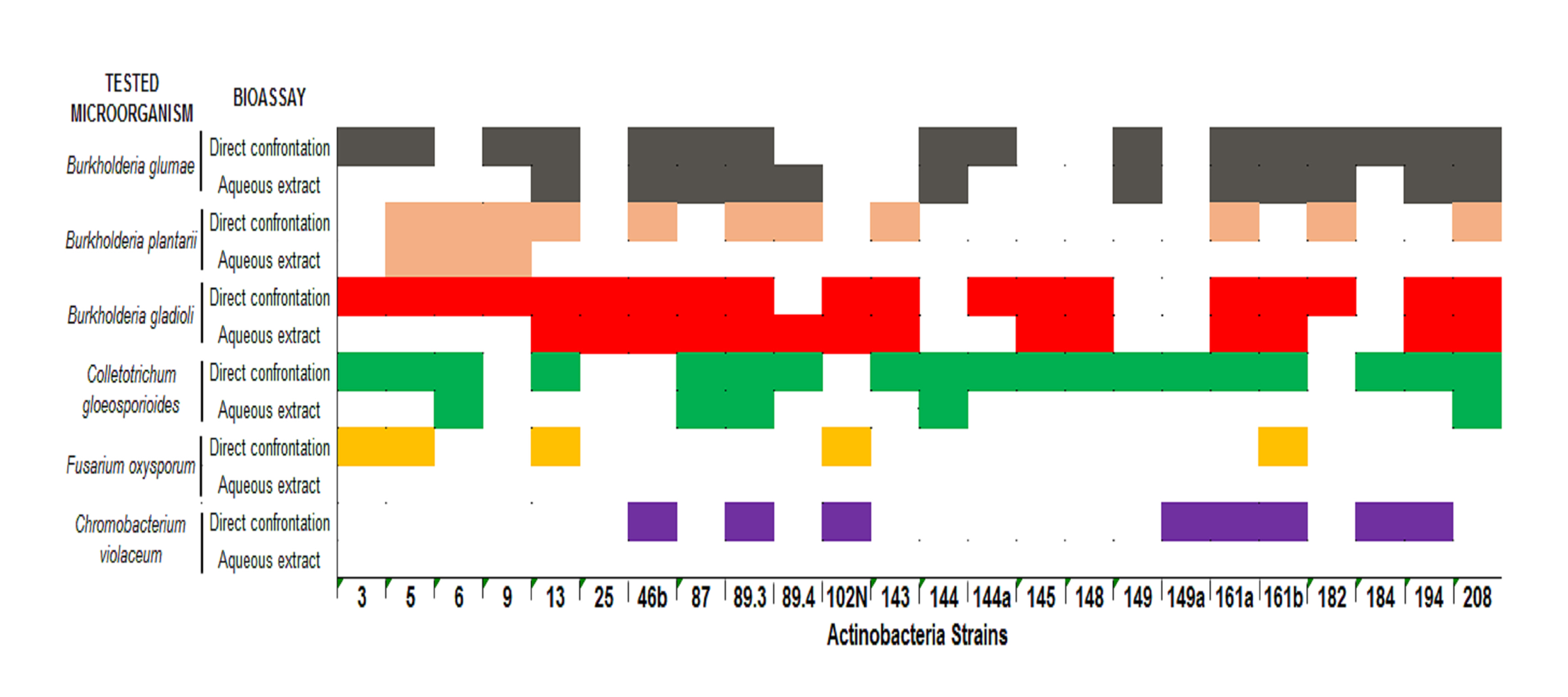

Supplement: S5 Fig — Heat map of bioactivity for the 24 strains and their aqueous extracts. Results for antibacterial, antifungal and QQ activities, are summarized. Horizontal axis shows the codes of the 24 Actinobacteria and vertical axis shows each one of the pathogens tested in both, direct confrontation and extract growth inhibition test. Color indicates the total or partial control of each phytopathogen, assumed as a positive result. (TIF) [file pone.0170148.s005.tif]

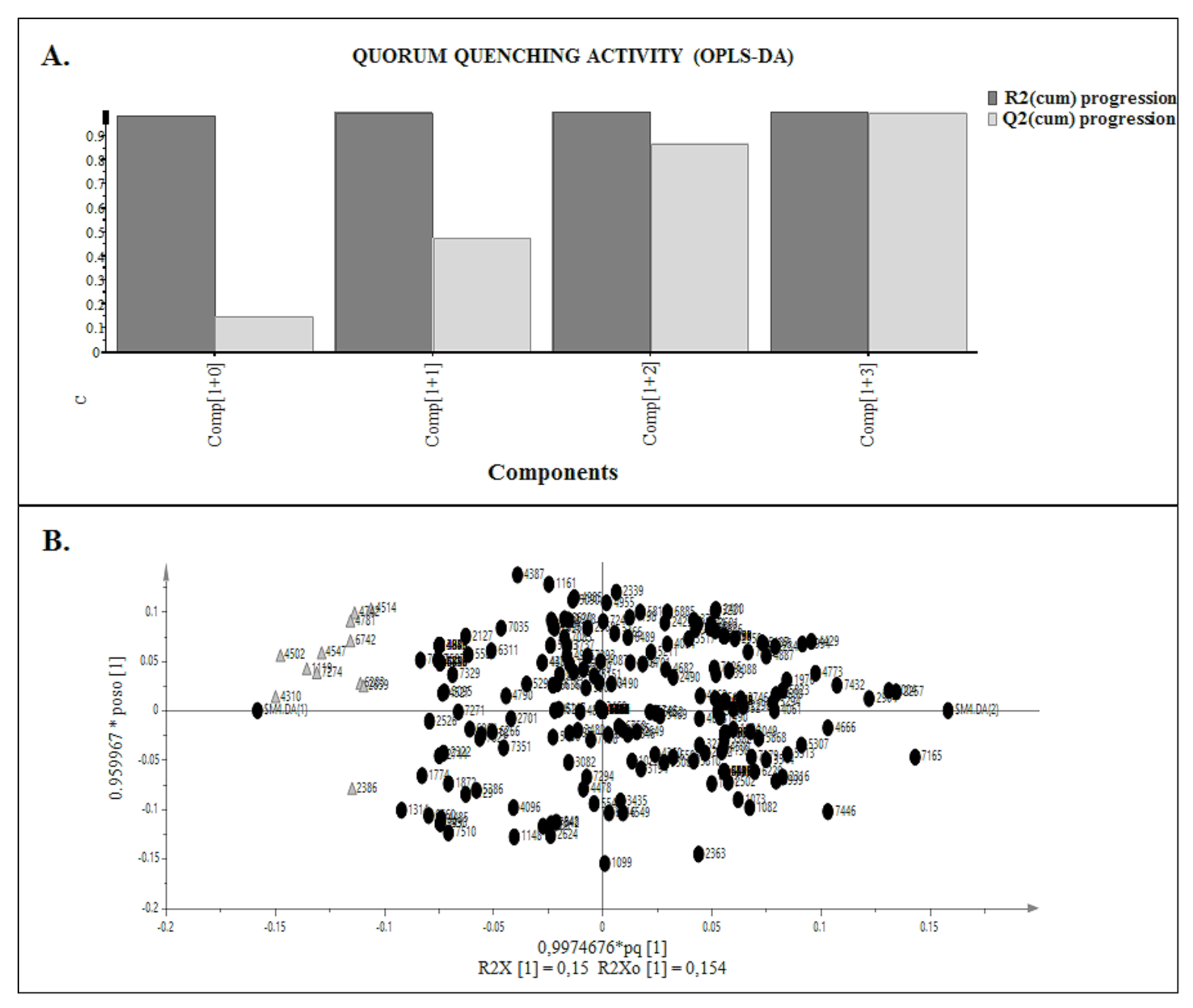

Supplement: S6 Fig — Summary of Fit (R2Y = 1, Q2 = 0.998). Loading Scatter Plot (VIPs, gray color). Strains 9, 46b, 87, 89.4, 102N, 184, 194. In this case data scaling was performed by unit variance (UV-) scaling. (TIF) [file pone.0170148.s006.tif]
