# Supplementary material for: Marine Actinobacteria as a source of compounds for phytopathogen control: An integrative metabolic-profiling / bioactivity and taxonomical approach
Source: PLoS One. 2017 Feb 22;12(2):e0170148. doi: 10.1371/journal.pone.0170148 (PMC5321270; doi:10.1371/journal.pone.0170148)
Supplement: S1 Table — API 20E Test (bioMérieux Inc., Durham, NC)–Oxidase and Catalase–L,L DAP (Actinobacteria). The test shows the presence of: ONPG: β-galactosidase; ADH: arginine-dihydrolase; LDC: lysine decarboxylase; ODC: ornithine decarboxylase. CIT: citrate utilization. Production of: H2S, URE: Urease, TDA: tryptophan deaminase, IND: indole, VP: acetoin, GEL: gelatinase. Other biochemical characteristics such as fermentation or oxidation of sugars (GLU: glucose, MAN: manitol, INO: inositol, SOR: sorbitol, RHA: rhamnose, SAC: sucrose, MEL: melibiose, AMY:amygdalin and ARA:arabinose It also shows the reduction of nitrates to nitrites. (PDF) [file pone.0170148.s007.pdf]

Table S1: Biochemical test for 24 marine strains. API 20E Test (bioMérieux Inc., Durham, NC) – Oxidase and Catalase – L,L DAP (*Actinobacteria*). The test shows the presence of: **ONPG**: β-galactosidase; **ADH**: arginine-dihydrolase; **LDC**: lysine decarboxylase; **ODC**: ornithine decarboxylase. **CIT**: citrate utilization. Production of: **H_2S**, **URE**: Urease, **TDA**: tryptophan deaminase, **IND**: indole, **VP**: acetoin, **GEL**: gelatinase. Other biochemical characteristics such as fermentation or oxidation of sugars (**GLU**: glucose, **MAN**: manitol, **INO**: inositol, **SOR**: sorbitol, **RHA**: rhamnose, **SAC**: sucrose, **MEL**: melibiose, **AMY**:amygdalin and **ARA**:arabinose It also shows the reduction of nitrates to nitrites.

TEST/ Strain	ONPG	ADH	LDC	ODC	CIT	H_2S	URE	TDA	IND	VP	GEL	GLU	MAN	INO	SOR	RHA	SAC	MEL	AMY	ARA	NO_2	oxidase	catalase	L,L DAP
**143**	+	+	+	-	-	-	+	+	-	+	+	-	-	-	-	-	-	-	-	+	-	+	+	+
**144**	+	+	-	-	-	-	-	+	-	+	+	-	-	-	-	-	-	-	-	+	-	+	+	+
**161A**	+	+	-	-	+	-	+	+	-	+	+	-	-	-	-	-	-	-	-	+	+	-	+	+
**161B**	+	-	-	+	+	-	+	+	-	-	+	-	+	-	-	-	-	-	-	-	-	-	-	+
**25**	+	-	-	-	-	-	+	+	-	+	+	-	+	+	-	-	-	+	-	+	+	-	+	-
**184**	+	-	-	-	-	-	+	+	-	-	+	+	-	-	-	-	-	-	-	-	-	-	+	+
**208**	+	-	-	+	-	-	+	+	-	+	+	-	-	-	-	-	-	-	-	+	-	-	+	+
**102N**	-	+	+	-	-	+	-	+	-	+	+	+	-	-	-	-	+	+	+	+	+	-	+	-
**13**	-	+	-	-	-	-	+	+	-	+	+	+	+	-	-	-	-	-	-	+	-	-	+	+
**144A**	-	+	-	-	-	-	-	+	-	+	+	-	-	-	-	-	+	-	-	-	-	+	+	+

**148**	-	+	-	+	-	-	-	+	-	-	+	+	-	-	-	-	-	-	-	+	+	-	+	+
**149**	-	+	-	-	-	-	-	+	-	+	+	-	-	-	-	-	+	-	-	+	+	+	+	+
**149A**	-	+	-	-	+	-	-	+	-	+	+	+	-	-	-	-	+	+	-	+	-	+	+	+
**87**	-	-	+	-	+	-	-	+	-	+	+	-	+	-	-	-	-	-	-	+	-	-	+	+
**89.3**	-	-	+	-	+	-	-	+	-	+	+	+	+	+	+	+	+	+	-	+	+	+	+	+
**89.4**	-	-	+	-	+	-	-	+	-	+	+	+	+	+	+	+	+	+	-	+	+	-	+	+
**6**	-	-	-	-	-	-	+	+	-	+	+	+	+	-	-	-	-	-	+	+	-	-	+	+
**46b**	-	-	-	-	-	-	+	-	+	+	+	+	+	+	+	+	+	+	-	+	+	-	+	+
**194**	-	-	-	-	-	-	-	+	-	-	+	+	-	-	-	-	-	+	-	+	+	-	+	+
**3**	-	-	-	-	-	-	-	+	+	+	+	+	-	+	+	+	-	+	-	+	-	-	+	+
**182**	-	-	-	-	-	-	-	+	-	+	+	-	-	-	-	-	-	+	-	+	-	-	+	+
**145**	-	-	-	-	-	-	-	+	-	+	+	-	-	-	-	-	-	-	-	+	-	+	+	+
**5**	-	-	-	-	-	-	-	+	-	+	+	+	+	+	+	+	-	+	-	+	-	+	+	+
**9**	-	-	-	-	-	-	-	+	-	+	+	+	+	+	+	+	-	+	-	+	-	+	+	+
