# Supplementary material for: Marine Actinobacteria as a source of compounds for phytopathogen control: An integrative metabolic-profiling / bioactivity and taxonomical approach
Source: PLoS One. 2017 Feb 22;12(2):e0170148. doi: 10.1371/journal.pone.0170148 (PMC5321270; doi:10.1371/journal.pone.0170148)

Table S2: Dereplication data of 36 variables of Strain 208 (Antimarin database)

m/z	RT	Molecular formula	Hits (Number of Hits from Antimarin-Antibase)	Activity
227.2	15.5	$C_{13}H_{10}N_2O_2$	**1-Hydroxy-6-methoxy-phenazine; 6-Methoxy-1-phenazinol**<br>**Source: *Streptomyces thioluteus***<br>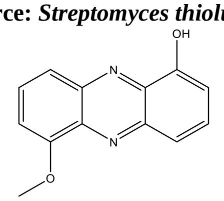	
		$C_{10}H_{14}N_2O_4$	**Nikkomycin D**<br>**Source: *Streptomyces tendae Tue 901***<br>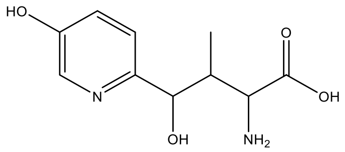	Peptidyl nucleoside antibiotic
		$C_{11}H_{14}O_5$	**Xialenone C**<br>**Source: *Streptomyces sp. GT 061169***<br>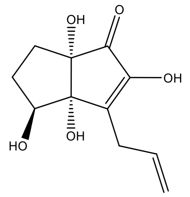	
		$C_{13}H_{10}N_2O_2$	**beta-Carboline-3-carboxylic acid methyl ester**<br>**Source: *Streptomyces sp. Ma5373***<br>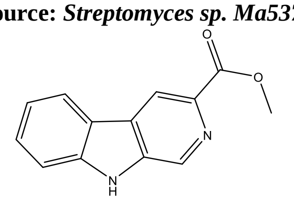	
**245.2**	**35.2**		**No hits**	
245.3	43.7	$C_{13}H_{24}O_4$	**4,5-Dihydro-4-hydroxymethyl-3-(1-hydroxy-6-methylheptyl)-2(3H)-furanone;**	Antibiotic

ST 43683;2-(6'-Methylheptanol-1'-yl)-3-hydroxymethyl-4-butanolide; Graefe's; Factor I (69.70)

Source*: Streptomyces viridochromogenes, S. cyaneofuscatus, S. viridochromogenes zimet, S. bikinensis ja 8031, S.cyaneofuscatus zimet*

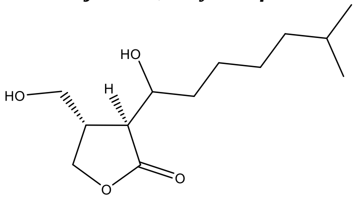

$C_{12}H_{20}O_5$ **YF-0200-R-B** Antibiotic

Source: ***Streptomyces sp. YF-0200R***

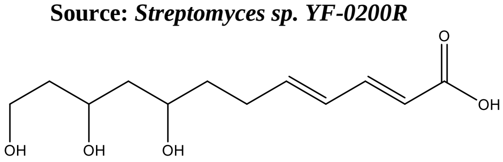

$C_{13}H_{24}O_4$ **Graefe's Factor 2**

Source: ***Streptomyces sp.***

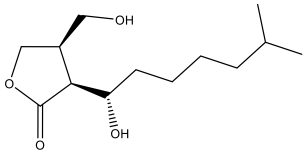

$C_{13}H_{24}O_4$ **SCB-1; (2R,3R,1'R)-2-(1'-Hydroxy-6-methylheptyl)-3- hydroxymethylbutanolide**

Source: ***Streptomyces coelicolor A3(2)***

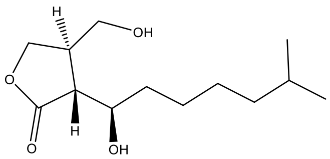

$C_{13}H_{24}O_4$ Antibiotic

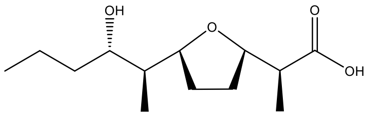

(71)

Trihomononactic acid

Source: ***Streptomyces globisporus***

$C_{11}H_{20}N_2O_4$ **Leucyl-4-hydroxyproline**

Source : ***Streptomyces acrimycini***

			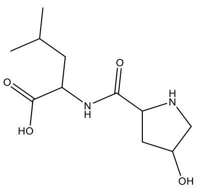	
**255.2**	**37.7**		**No hits**	
255.3	19.5	$C_{14}H_{10}N_2O_3$	**N-Acetylquestiomycin A;**<br>**2-Acetamido(3H)phenoxazin-3-one**<br>**Source:** ***Streptomyces thioluteus, Microbispora***<br>***aerate***<br>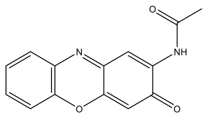	(73,74)
		$C_{15}H_{10}O_4$	**Daidzein; Isoaurostatin; K-251b;**<br>**NPI-031E**<br>**Source: GW82/2497**<br>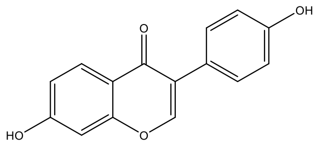	
		$C_{15}H_{10}O_4$	**Aloesaponarin II**<br>**Source:** ***Streptomyces sp. GW 24/1694, L083***<br>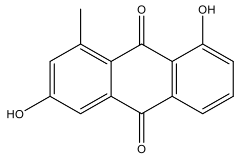	Antibiotic
		$C_{14}H_{10}N_2O_3$	**1-Hydroxymethyl-6-carboxyphenazine**<br>**Source:** ***Streptomyces griseoluteus; marine Vibrio sp. SANK 73794***<br>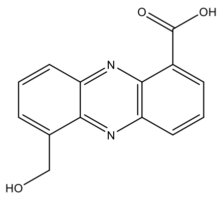	Antibiotic
273.3	7.33	$C_{13}H_8N_2O_5$	**2,3,9-Trihydroxyphenazine-1-carboxylic acid**	

Source: ***Pseudomonas fluorescens***

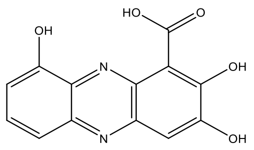

$C_{14}H_8O_6$ **Aplidioxin B**

Source: ***Aplidiopsis ocellata***

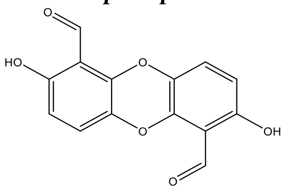

288.3 39.2 $C_{10}H_{13}N_3O_7$ **Nikkomycin Cx** Antibiotic

Source: ***Streptomyces tendae Tue 901 (ATCC 31160, Cbs 354.75, FERM-p 3136)***

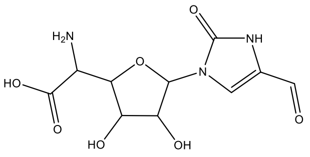

$C_{10}H_{13}N_3O_7$ **Nikkomycin C; Nikkomycin Cz**

Source: ***Streptomyces tendae Tue 901 (ATCC 31160, Cbs 354.75, FERM-p 3136)***

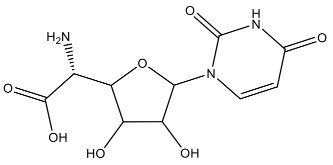

300.1 **37.5** **No hits**

337.2 **6.4** **No hits**

337.4 42.2 $C_{19}H_{12}O_6$ **WS 5995-A** Antibiotic

Source: ***Streptomyces auranticolor 5995 (FERM-p 5365)***

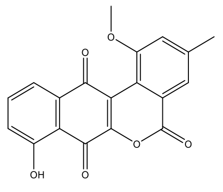

$C_{19}H_{12}O_6$ **Tetracenomycin D1; 9-Decarboxy-tetracenomycin-F1** Cytotoxic

	**Source:** ***Streptomyces glaucescens Tue 49 (eth 22794); marine Streptomycete B-8005*** 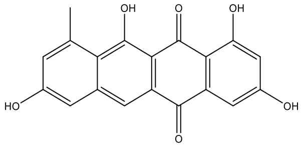	Anticancer<br>Antibiotic<br>Antibacterial
$C_{20}H_{16}O_5$	**Fujianmycin B; Rubiginone A2; SNA-8073-A**<br>**Source:** ***Streptomyces sp.*** 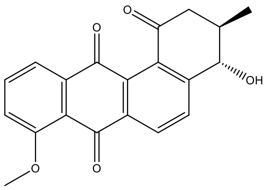	Antibiotic and antibacterial compound
$C_{20}H_{16}O_5$	**6-Deoxy-8-O-methylrabelomycin; MM 47755; 8-O-Methyltetrangomycin**<br>**Source:** ***Streptomyces sp. GW11/3289*** 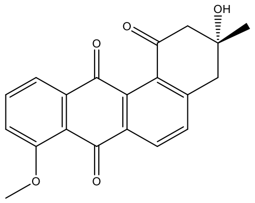	Antibiotic agent. Shows antibacterial and antifungal effects
$C_{20}H_{16}O_5$	**Hatomarubigin-A; CE-33-A**<br>**Source*****: Streptomyces sp. 2238-svt4 (FERM bp-3326*** 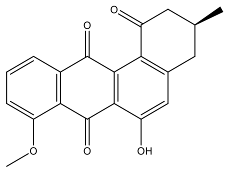	Antibiotic
$C_{20}H_{16}O_5$	**Hatomarubigin-B; CE-33-B**<br>**Source:** ***Streptomyces sp. 2238-svt4*** 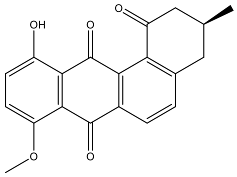	Antibiotic

		$C_{20}H_{16}O_5$	**SNA-8073-B**<br>**Source *Streptomyces sp. SNA-8073***<br>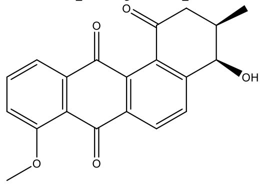	Antibiotic
		$C_{19}H_{12}O_6$	**6,9,11-Trihydroxy-4-methoxy-5,12-naphthacendione**<br>**Source: *Streptomyces sp. GW37/3236***<br>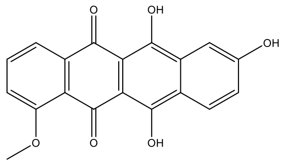	
339.3	15.7	$C_{19}H_{14}O_6$	**WS 5995B**<br>**Source: *Streptomyces auranticolor 5995 (FERM-p 5365), Streptomyces sp. AcH***<br>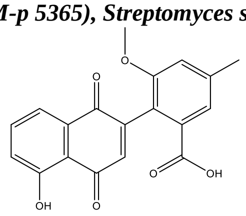	Antibiotic<br>Inhibits colonisation by the root and butt rot fungus Heterobasidion annosum 005<br>Inhibits Norway spruce root infection
		$C_{19}H_{14}O_6$	**Rabelomycin; Fridamycin C**<br>**Source: S. *tsusimaensis, S.olivaceus, marine***<br>***Streptomyces spp. B 6921, B6935, B6728***<br>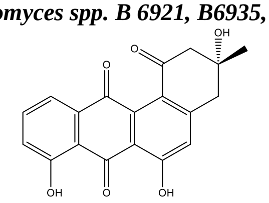	Antibiotic
		$C_{19}H_{14}O_6$	**Landomycinone A**<br>**Source: Streptomyces sp**<br>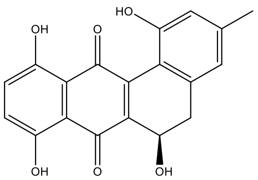	Antibiotic

$C_{19}H_{14}O_6$ **Lagumycin A**

Source: marine *Streptomyces sp. B 8245*

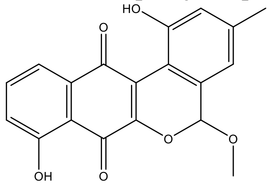

359.3 32.2 $C_{13}H_{14}N_2O_{10}$ **Octosyl acid-A**

Source: *Streptomyces cacaoiasoensis*

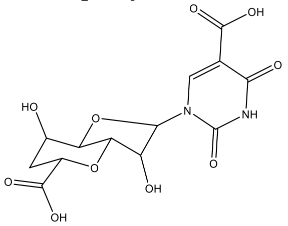

$C_{19}H_{18}O_7$ **2-Deoxy-5,6-dihydro-PD-116198**

Source : *Streptomyces phaeochromogenes WP 3688* Antibiotic

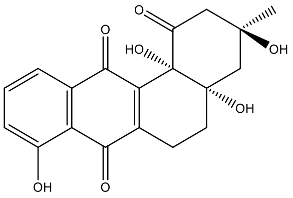

$C_{19}H_{18}O_7$ **Spectomycin-A2** Antibiotic

Source: *Streptomyces spectabilis*

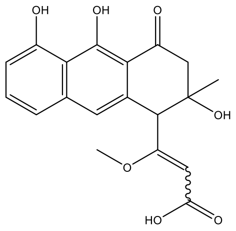

$C_{19}H_{18}O_7$ **Elmycin-C** Antibiotic

Source: *Streptomyces sp.*

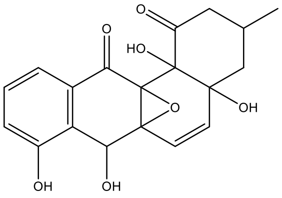

		$C_{19}H_{18}O_7$	**Simocyclinon A1**<br>**Source:** ***Streptomyces sp. Tue 6040***<br>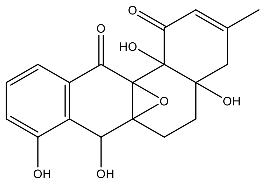	Antibacterial and antitumor properties
		$C_{19}H_{18}O_7$	**4,4a,8,9,10,12b-Hexahydro-4a,7,8,12,113a,-pentahydroxy--3-methylbenz[a]anthracene-1,11-dione**<br>**Source:** ***Streptomyces sp. 1B1***<br>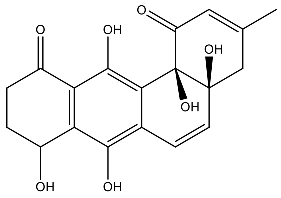	
		$C_{19}H_{18}O_7$	**Panglimycin D**<br>**Source:** ***Streptomyces sp. ICBB8230***<br>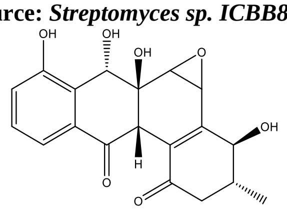	
**381.1**	**36.4**		**No hits**	
**383.0**	**37.4**		**No hits**	
**392.1**	**37.0**		**No hits**	
**395.2**	**37.2**		**No hits**	
**423.2**	**35.3**		**No hits**	
**425.1**	**40.5**		**No hits**	
**453.0**	**15.7**		**No hits**	
**455.1**	**31.2**		**No hits**	
**457.2**	**9.3**		**No hits**	
**464.0**	**37.2**		**No hits**	
**475.0**	**11.9**		**No hits**	

**482.3**	**5.6**		**No hits**	
**484.0**	**37.5**		**No hits**	
**494.1**	**31.7**		**No hits**	
**495.1**	**37.0**		**No hits**	
**500.1**	**36.8**		**No hits**	
**505.0**	**35.0**		**No hits**	
**516.2**	**39.5**		**No hits**	
**526.1**	**36.5**		**No hits**	
**563.3**	**8.4**		**No hits**	
**639.1**	**40.5**		**No hits**	
**684.7**	**43.4**		**No hits**	
**714.3**	**40.0**		**No hits**	
**775.3**	**44.4**		**No hits**	
262.4	30.7	$C_{16}H_{23}NO_2$	**Name: Trichostatinol**<br>**Source: *Streptomyces* sp. GW37/655**<br>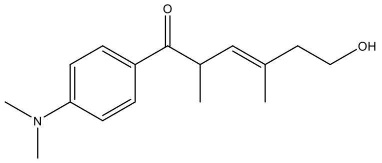	
540.5	9.2	$C_{30}H_{21}NO_9$	**Name: Fredericamycin-A; FCRC-A-48; FCRC-A-48-A; NSC-305263**<br>**Source**: ***Streptomyces griseus (fcrc-48)***<br>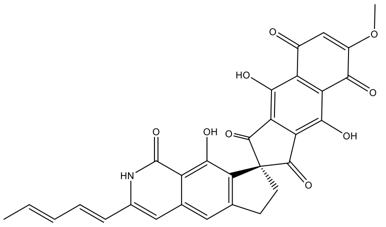	Antibiotic<br>(72)
415.5	37.1	$C_{21}H_{38}N_2O_6$	**Name:NK-04000Q; Epopromycin B**	

Source: ***Streptomyces*** **sp. NK04000**

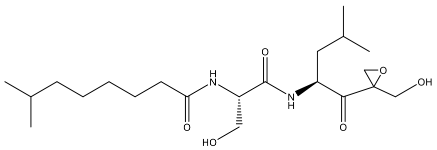

$C_{22}H_{38}O_7$ **Name: Feigrisolide D** Macrolide

Source: ***Streptomyces griseus*** (75)

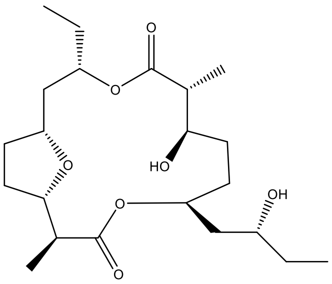

446.6 40.9 $C_{26}H_{39}NO_5$ **Name: Piericidin-C2; Piericidin-F** **Anthelmintic activity**

Source: ***Streptomyces piericidicus, S.pactum*** (67,68)

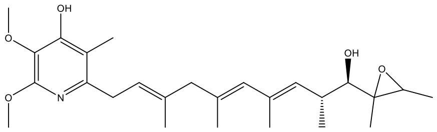

Supplement: S2 Table — (PDF) [file pone.0170148.s008.pdf]
